# Fluc‐EGFP reporter mice reveal differential alterations of neuronal proteostasis in aging and disease

**DOI:** 10.15252/embj.2020107260

**Published:** 2021-08-19

**Authors:** Sonja Blumenstock, Elena Katharina Schulz‐Trieglaff, Kerstin Voelkl, Anna‐Lena Bolender, Paul Lapios, Jana Lindner, Mark S Hipp, F Ulrich Hartl, Rüdiger Klein, Irina Dudanova

**Affiliations:** ^1^ Department of Molecules – Signaling – Development Max Planck Institute of Neurobiology Martinsried Germany; ^2^ Molecular Neurodegeneration Group Max Planck Institute of Neurobiology Martinsried Germany; ^3^ Department of Cellular Biochemistry Max Planck Institute of Biochemistry Martinsried Germany; ^4^ Department of Biomedical Sciences of Cells and Systems University Medical Center Groningen University of Groningen Groningen The Netherlands; ^5^ School of Medicine and Health Sciences Carl von Ossietzky University Oldenburg Oldenburg Germany

**Keywords:** Huntington’s disease, nuclear and cytoplasmic aggregates, protein homeostasis, reporter mouse, tauopathy, Neuroscience, Post-translational Modifications & Proteolysis

## Abstract

The cellular protein quality control machinery is important for preventing protein misfolding and aggregation. Declining protein homeostasis (proteostasis) is believed to play a crucial role in age‐related neurodegenerative disorders. However, how neuronal proteostasis capacity changes in different diseases is not yet sufficiently understood, and progress in this area has been hampered by the lack of tools to monitor proteostasis in mammalian models. Here, we have developed reporter mice for *in vivo* analysis of neuronal proteostasis. The mice express EGFP‐fused firefly luciferase (Fluc‐EGFP), a conformationally unstable protein that requires chaperones for proper folding, and that reacts to proteotoxic stress by formation of intracellular Fluc‐EGFP foci and by reduced luciferase activity. Using these mice, we provide evidence for proteostasis decline in the aging brain. Moreover, we find a marked reaction of the Fluc‐EGFP sensor in a mouse model of tauopathy, but not in mouse models of Huntington’s disease. Mechanistic investigations in primary neuronal cultures demonstrate that different types of protein aggregates have distinct effects on the cellular protein quality control. Thus, Fluc‐EGFP reporter mice enable new insights into proteostasis alterations in different diseases.

## Graphical abstract

*In vivo* expression of conformationally unstable EGFP‐fused firefly luciferase, which forms intracellular foci and exhibits reduced activity upon proteotoxic stress, elucidates the occurrence of proteostasis decline in mouse models of aging and tauopathy but not Huntington's disease.

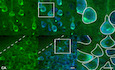

## Introduction

Maintaining the integrity of the cellular proteome is essential for survival. The cellular protein quality control system safeguards protein homeostasis (proteostasis) by ensuring correct folding of new proteins, detecting and refolding damaged proteins, and targeting terminally misfolded proteins for degradation (Balchin *et al*, 2016; Klaips *et al*, 2018). Age‐dependent decline in protein quality control is believed to play a crucial role in neurodegenerative diseases, a group of brain disorders characterized by aggregation of misfolded proteins and neuronal cell death, such as Alzheimer’s, Parkinson’s, and Huntington’s disease (HD; Soto & Pritzkow, 2018). Enhancing the capacity of the protein quality control system has therefore emerged as a promising therapeutic strategy for neurodegenerative proteinopathies (Smith *et al*, 2015; Klaips *et al*, 2018). However, our current knowledge about the proteostasis changes *in vivo* during disease progression is still scarce. While attempts to ameliorate aggregate toxicity by upregulating chaperones have been successful in cell culture, fly, and worm models (Carmichael *et al*, 2000; Auluck *et al*, 2002; Outeiro *et al*, 2006; Hageman *et al*, 2010; Vos *et al*, 2010; Wu *et al*, 2010; Kuo *et al*, 2013), they have produced less satisfactory results in mammalian models (Hansson *et al*, 2003; Liu *et al*, 2005; Zourlidou *et al*, 2007; Krishnan *et al*, 2008; Sharp *et al*, 2008; Shimshek *et al*, 2010; Labbadia *et al*, 2012; Xu *et al*, 2015). Reliable genetic reporters that allow monitoring the status of cellular proteostasis *in vivo* are essential for understanding disease mechanisms and for assessing the efficacy of potential treatments targeting the protein quality control system. Thus, transgenic mice expressing ubiquitin‐proteasome system (UPS) reporters have been used successfully for investigating protein degradation in disease models (Lindsten *et al*, 2003; Kristiansen *et al*, 2007; Bett *et al*, 2009; Cheroni *et al*, 2009; Ortega *et al*, 2010; Myeku *et al*, 2016). However, tools for monitoring proteostasis in general are still lacking.

Wild‐type and mutated versions of the conformationally unstable firefly luciferase (Fluc) protein fused to EGFP are ideal for use as proteostasis sensors and have proven valuable in cell lines and in *C. elegans* (Gupta *et al*, 2011; Donnelly *et al*, 2014). These sensors depend on cellular chaperones for proper folding and enzymatic activity. Proteotoxic conditions that overload the protein quality control system lead to misfolding of Fluc‐EGFP, which can be revealed by two readouts: decrease in bioluminescence due to decline in luciferase activity and formation of Fluc‐EGFP foci in the cell as a result of decreased solubility of the misfolded sensor (Gupta *et al*, 2011).

To gain a deeper understanding of proteostasis changes in aging and disease, we generated new reporter mice expressing Fluc‐EGFP in the nervous system. Using these mice, we reveal unexpected differences in proteostasis alterations caused by different types of protein aggregates.

## Results

### Fluc‐EGFP sensor reacts to proteostasis changes in primary neurons

**Figure EV1:**
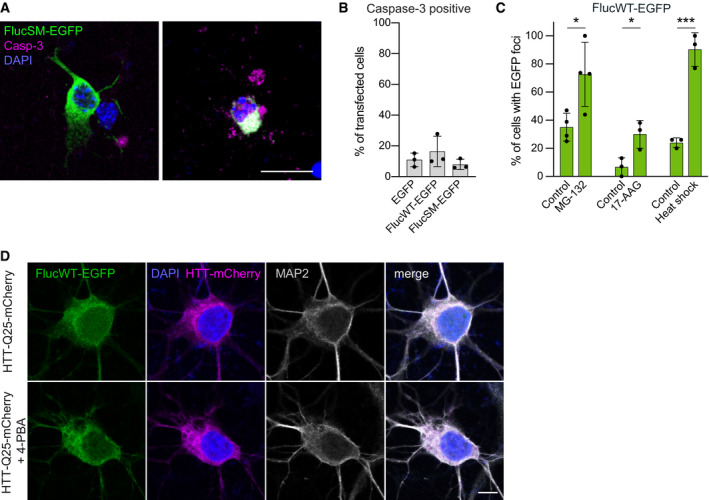
Additional experiments with Fluc‐EGFP in primary neurons Examples of neurons expressing FlucSM‐EGFP (green) that are negative (left) or positive (right) for cleaved caspase‐3 (magenta). Nuclei are labeled with DAPI (blue).Quantification of the fraction of transfected neurons positive for cleaved caspase‐3 at DIV 3 + 2. *N* = 3 independent experiments; one‐way ANOVA. No significant differences were observed.Quantification of FlucWT‐EGFP foci formation in transfected neurons upon indicated treatments. *N* = 3–4 independent experiments; two‐tailed *t*‐test.DIV 3 + 2 cortical neurons co‐transfected with FlucWT‐EGFP (green) and HTT‐Q25‐mCherry (magenta) and treated with 4‐PBA (lower row) or vehicle control (upper row) from DIV 3. Cultured neurons were stained for MAP2 (gray) as a neuronal marker, and nuclei were labeled with DAPI (blue). Data information: Error bars represent SD. Significance: *P < 0.05; ***P < 0.001. Scale bars: A, 20 µm; D, 5 µm.

**Figure 1:**
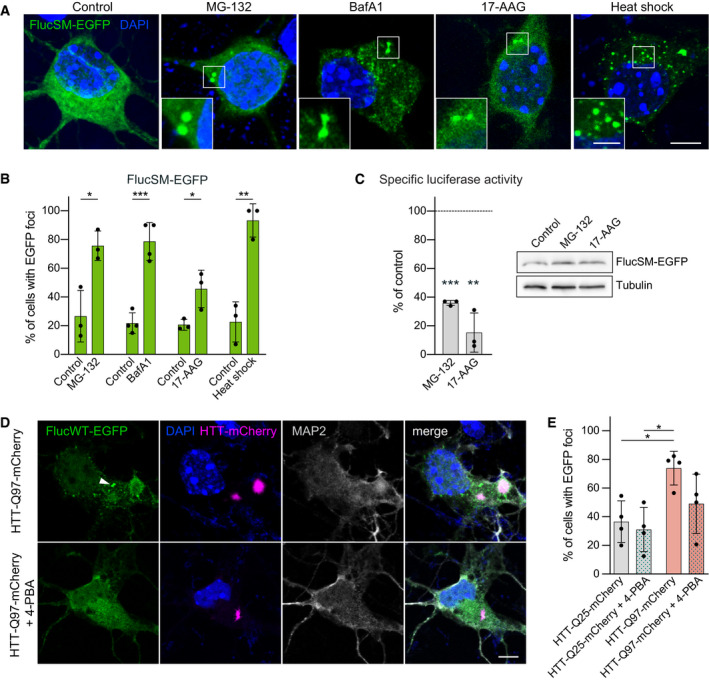
Fluc‐EGFP reacts to proteostasis changes in primary neurons Representative images of DIV 3 + 2 cortical neurons transfected with FlucSM‐EGFP (green) and subjected to the indicated treatments: 5 µM MG‐132 for 4 h; 10 nM Bafilomycin A1 (BafA1) for 24 h; 0.5 µM 17‐AAG for 4 h; heat shock at 43°C for 30 min. Nuclei were labeled with DAPI (blue). Insets show higher magnification of the areas outlined by the boxes.Quantification of FlucSM‐EGFP foci formation in transfected neurons. *N* = 4 biological replicates for BafA1 and corresponding control group, 3 biological replicates for all the other conditions; two‐tailed *t*‐test.Left, quantification of specific luciferase activity of FlucSM‐EGFP upon indicated treatments, normalized to respective vehicle‐treated controls. *N* = 3 biological replicates; one‐sample *t*‐test. Right, representative Western blot of neuronal lysates for the indicated conditions. Tubulin was used as a loading control.DIV 3 + 2 cortical neurons co‐transfected with FlucWT‐EGFP (green) and HTT‐Q97‐mCherry (magenta) and treated with 1 mM 4‐PBA (lower row) or vehicle control (upper row) from DIV 3. Cells were stained for MAP2 (gray) as a neuronal marker, and nuclei were labeled with DAPI (blue). Arrowhead points to Fluc‐EGFP foci. Corresponding cultures transfected with control HTT are shown in Fig EV1D.Quantification of the fraction of double‐transfected cells showing Fluc‐EGFP foci. *N* = 4 biological replicates. Two‐way ANOVA with Tukey’s multiple comparisons test. ANOVA: HTT, **P = 0.0046; 4‐PBA, n.s.; HTT × 4‐PBA, n.s. Significant pairwise comparisons are indicated on the graph. Data information: Error bars represent SD. Significance: *P < 0.05, **P < 0.01, ***P < 0.001. Scale bars: A and D, 5 µm; insets in A, 2 µm. Source data are available online for this figure.

We first asked whether Fluc‐EGFP variants can be used as reporters of proteotoxic stress in primary neurons. In the following experiments, we used two versions of Fluc, wild‐type (FlucWT) and single mutant FlucR188Q (FlucSM). FlucSM is a conformationally destabilized mutant that was previously shown to have higher sensitivity to proteotoxic stress than FlucWT (Gupta *et al*, 2011), however, its expression levels in neurons were relatively low, possibly due to efficient degradation. Transfection of Fluc‐EGFP constructs did not lead to toxicity in murine primary cortical cultures, as demonstrated by immunostaining against the apoptotic marker cleaved caspase‐3 (Fig EV1A and B). Cultures transfected with Fluc‐EGFP constructs were subjected to several treatments to induce proteotoxic stress. MG‐132 was used to inhibit protein degradation by the proteasome, Bafilomycin A1 was used to inhibit autophagy, and 17‐AAG was used to inhibit Hsp90, a major cytosolic chaperone (Appendix Fig S1A–C). All these treatments induced a change in the distribution of Fluc‐EGFP, which typically formed several small compact foci in the perinuclear region, while the intensity of diffuse EGFP fluorescence in the rest of the cytoplasm decreased (Figs 1A and B, and Fig EV1C). In addition, cells were subjected to heat shock at 43°C, a treatment known to induce proteotoxic stress (Nishimura *et al*, 1991; Yang *et al*, 2008; Morimoto, 2011). Heat shock led to an even stronger Fluc‐EGFP response, with many cells showing a complete loss of diffuse cytoplasmic EGFP fluorescence and formation of multiple Fluc‐EGFP foci throughout the cytoplasm (Figs 1A and B, and Fig EV1C).

We used the luciferase assay to evaluate the enzymatic activity of Fluc‐EGFP as an additional readout of proteostasis alterations. Throughout the study, luciferase activity measurements were normalized to Fluc‐EGFP protein quantity determined by Western blot to obtain specific activity values. As expected, proteasome inhibition with MG‐132 and Hsp90 inhibition with 17‐AAG both resulted in a significant decrease in specific luciferase activity of FlucSM‐EGFP by ∼65% and ∼85%, respectively (Fig 1C). Overall, the response of Fluc‐EGFP to heat shock and small‐molecule inhibitors in primary neurons appeared stronger than the response observed in non‐neuronal cell lines (Gupta *et al*, 2011), probably due to the high sensitivity of neurons to proteotoxic stress.

We next asked whether Fluc‐EGFP reacts to the proteostasis dysbalance caused by an aggregating protein. To this end, we co‐transfected primary neurons with Fluc‐EGFP and the pathologically expanded form of mutant Huntingtin (mHTT)‐exon1 (HTT‐Q97‐mCherry). mHTT‐exon1 is a key pathogenic version of the protein that is sufficient to recapitulate HD phenotypes (Mangiarini *et al*, 1996; Sathasivam *et al*, 2013; Yang *et al*, 2020). Co‐transfection with HTT‐Q97‐mCherry resulted in a significant increase in Fluc‐EGFP foci compared to control cells co‐transfected with HTT‐Q25‐Cherry (Figs 1D and E, and Fig EV1D). To ensure that the changes in Fluc‐EGFP solubility were proteostasis‐dependent, we treated the cultures with the chemical chaperone 4‐phenylbutyrate (4‐PBA). 4‐PBA has been shown to counteract protein misfolding and aggregation in several proteinopathy models (Yam *et al*, 2007; Wiley *et al*, 2011; Winter *et al*, 2014; Hirata *et al*, 2020). The frequency of Fluc‐EGFP foci in HTT‐Q97‐mCherry cells treated with 4‐PBA was not significantly different from that in control HTT‐Q25‐mCherry cells (Fig 1D and E), suggesting that formation of Fluc‐EGFP foci is indeed due to protein misfolding. Taken together, these results demonstrate that Fluc‐EGFP can be reliably used to detect proteostasis disturbances in primary neurons.

### Fluc‐EGFP reporter mouse for *in vivo* analysis of proteostasis

**Figure 2:**
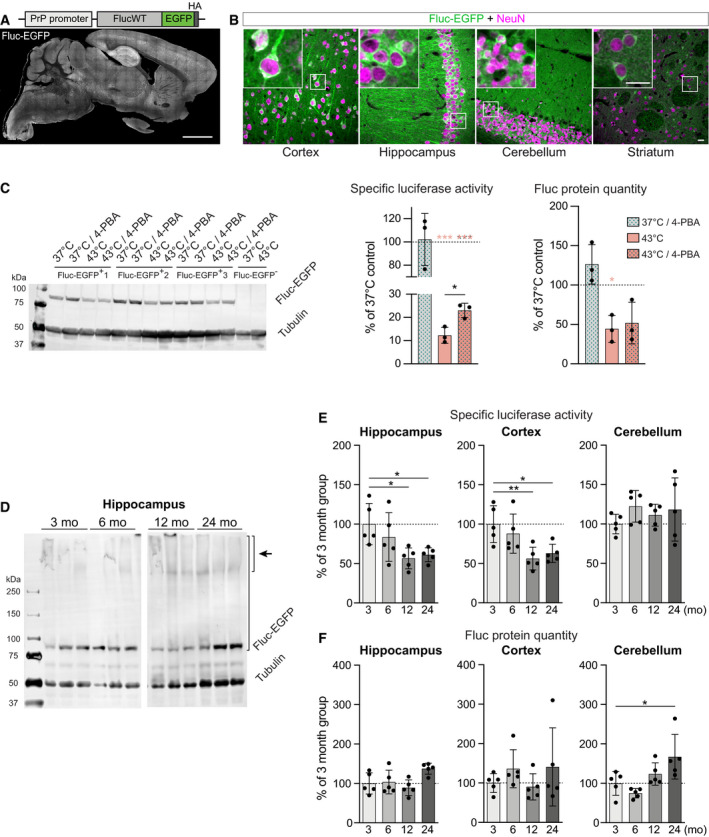
Fluc‐EGFP reporter reveals proteostasis impairment in aging mice Scheme of the transgenic construct (top) and sagittal brain section of a Fluc‐EGFP mouse from line 1,214 at 3 months of age immunostained for EGFP (bottom).Representative images of the indicated brain regions of a Fluc‐EGFP mouse, stained for EGFP (green) and the neuronal marker NeuN (magenta). Insets show higher magnification of the areas indicated by the boxes.Left, Western blot of acute brain slice lysates from Fluc‐EGFP mice and non‐transgenic littermates. The slices were treated with 1 mM 4‐PBA or vehicle control and subjected to heat shock at 43°C for 15 min, as indicated above the blot. Middle, quantification of specific luciferase activity of Fluc‐EGFP upon indicated treatments, normalized to vehicle‐treated slices kept at 37°C. Right, quantification of Fluc‐EGFP protein levels in the indicated conditions. *N* = 3 mice. Colored asterisks indicate comparisons to the corresponding vehicle‐treated 37°C control group (one‐sample *t*‐test), black asterisk indicates comparison between 4‐PBA and vehicle‐treated heat shock groups (two‐tailed *t*‐test).Representative Western blot of hippocampal lysates from Fluc‐EGFP mice at the indicated ages. Short bracket with an arrow indicates the high molecular weight species observed in older mice. Long bracket indicates the part of the lane that was used for Fluc protein quantification (see Materials and Methods). Several lanes on the blot between 6‐month‐old and 12‐month‐old samples were digitally removed.Fluc‐EGFP specific luciferase activity measured in the indicated brain regions of Fluc‐EGFP mice at the indicated ages. Values are normalized to the 3‐month‐old group. *N* = 5 Fluc‐EGFP mice for each age group. One‐way ANOVA with Bonferroni’s multiple comparisons test. ANOVA: Hippocampus, *P = 0.0205; Cortex, **P = 0.0077; Cerebellum, n.s. Significant pairwise comparisons to the 3‐month‐old group are indicated on the graphs.Fluc‐EGFP protein quantity measured in the indicated brain regions of Fluc‐EGFP mice at the indicated ages. Values are normalized to the 3‐month‐old group. *N* = 5 Fluc‐EGFP mice for each age group. One‐way ANOVA with Bonferroni’s multiple comparisons test. ANOVA: Hippocampus, *P = 0.0278; Cortex, n.s; Cerebellum, **P = 0.0058. Significant pairwise comparisons to the 3‐month‐old group are indicated on the graphs. Protein quantity normalized to total protein instead of tubulin is shown in Appendix Fig S2C. Data information: Error bars represent SD. Significance: *P < 0.05; **P < 0.01, ***P < 0.001. Scale bars: A, 2 mm; B, 20 µm. Source data are available online for this figure.

**Figure EV2:**
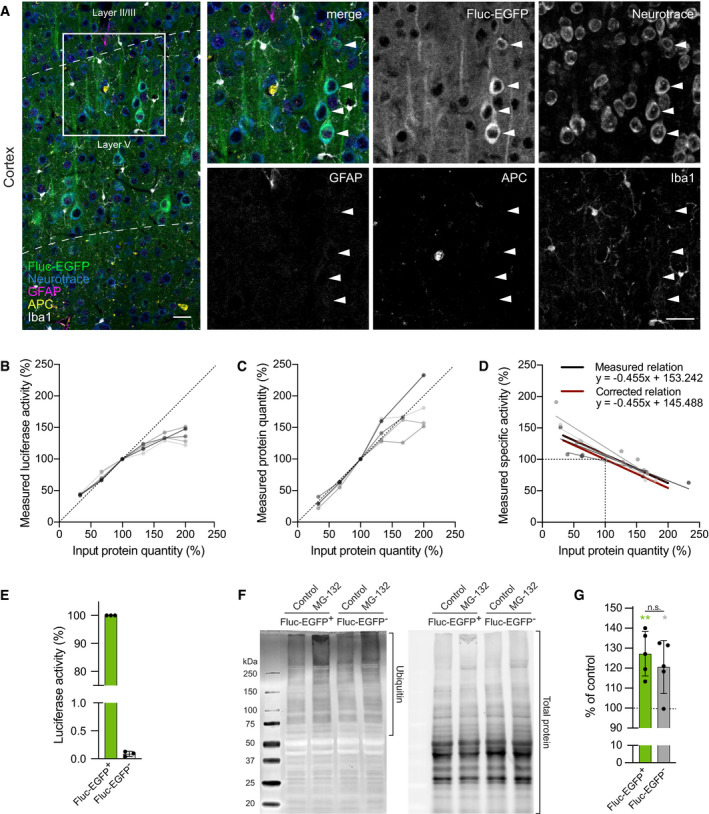
Characterization of Fluc‐EGFP reporter mice AAnalysis of Fluc‐EGFP expression in neurons and glia. Cortical sections from 3 to 4‐month‐old Fluc‐EGFP mice were stained against GFAP (magenta), APC (yellow), and Iba1 (white). Fluc‐EGFP was detected by EGFP fluorescence (green), and neurons were labeled with Neurotrace (blue). Arrowheads point to Fluc‐EGFP‐positive cells. Dashed lines indicate borders of cortical layers. The experiment was repeated in *N* = 5 animals with similar results. Similar analysis in the hippocampus is shown in Appendix Fig S2A.B–DLinearity of luciferase activity in tissue samples. Cortical lysates from 5 Fluc‐EGFP mice (shown in shades of gray) were used in different dilutions (25, 50, 75, 100, 125 and 150 μg input protein quantity). All values were normalized to the 75 μg dilution. (B) Measured luciferase activity of the samples. (C) Measured protein quantity. (D) Linear regression of input protein quantity to specific luciferase activity (measured luciferase activity normalized to measured protein quantity) for individual mice (gray lines) and mean regression of five mice (black line); red line shows the corrected relation where 100% input protein quantity corresponds to 100% specific activity.EQuantification of luciferase activity in Fluc‐EGFP mice and non‐transgenic littermates. *N* = 3 mice per genotype.FWestern blots of acute brain slices of indicated genotype treated with 5 µM MG‐132 or vehicle (control) for 4 h. The part of the lane used for quantification of ubiquitin is indicated with a bracket (left). Total protein load (right, stain‐free blot) was used for normalization.GQuantification of increase in ubiquitinated proteins upon MG‐132 treatment in brain slices from Fluc‐EGFP mice and control littermates. *N* = 5 mice of each genotype. Green and gray asterisks indicate comparisons to the corresponding vehicle‐treated control slices by one‐sample *t*‐test. Comparison between MG‐132 treated slices of different genotypes was performed by two‐tailed *t*‐test. Data information: Error bars represent SD. Significance: *P < 0.05; **P < 0.01; n.s.—not significant. Scale bars in A, 30 µm. Source data are available online for this figure.

For *in vivo* studies of the protein quality control system in mouse models, we generated transgenic mouse lines expressing FlucWT‐EGFP or FlucSM‐EGFP under the control of the prion protein (PrP) promoter (Fig 2A). In line with our observations in primary neurons, FlucSM‐EGFP mouse lines showed rather low expression of the sensor. For further experiments, we selected the FlucWT‐EGFP line 1,214 (from here on, Fluc‐EGFP mice), which had a broad expression of the transgene throughout the brain, including regions affected in neurodegenerative proteinopathies. In particular, stronger expression was detected in the neocortex and hippocampus, while lower levels were observed in the basal ganglia and cerebellum (Fig 2A and B). Co‐staining with cell type markers demonstrated that Fluc‐EGFP was present in Neurotrace^+^ neurons, while it was not detectable in GFAP^+^ astrocytes, APC^+^ oligodendrocytes, or Iba^+^ microglia (Fig EV2A and Appendix Fig S2A). In neurons, Fluc‐EGFP showed cytoplasmic localization in the soma and dendrites (Figs 2B and EV2A, and Appendix Fig S2A).

Unlike in cell culture conditions, the quantity of the Fluc‐EGFP protein might vary considerably between tissue samples, which could lead to a bias when using bioluminescence measurements *in vivo*. We therefore tested whether luciferase activity changes linearly to the Fluc‐EGFP protein concentration using serial dilutions of 5 cortical tissue samples from 2‐month‐old Fluc‐EGFP mice. We found that higher levels of Fluc‐EGFP resulted in lower bioluminescence values than expected (Fig EV2B and C). The relationship between Fluc‐EGFP protein quantity in the sample (*x*) and expected specific luciferase activity of the sample (*y*) was described by the formula: *y* = −0.45488*x* + 145.488 (Fig EV2D). All measurements of specific activity in tissue samples were therefore corrected accordingly. No luciferase activity was detected in non‐transgenic littermate controls (Fig EV2E).

As prolonged expression of an unstable protein such as Fluc might cause adaptive changes in the proteostasis system, we compared response to proteotoxic stress in Fluc‐EGFP mice and wild‐type littermates. Acute brain slices were treated with the proteasome inhibitor MG‐132 for 4 h, and levels of ubiquitinated proteins were determined in the slice lysates by Western blot (Fig EV2F and G). Of note, we observed time‐dependent decrease in Fluc‐EGFP protein levels in brain slices (Appendix Fig S2B), which was more prominent in heat shock conditions (Fig 2C and Appendix Fig S2B). All experiments with slices were therefore performed keeping incubation times as short as possible, and not exceeding 4 h. The increase in ubiquitinated proteins induced by MG‐132 was not different between Fluc‐EGFP and control mice (Fig EV2F and G). These data suggest that Fluc‐EGFP expression does not lead to a major change in the cellular proteostasis capacity in our transgenic mouse model.

To ensure that Fluc‐EGFP responds to proteostasis alterations also in brain tissue, we performed a proteostasis rescue experiment. Acute brain slices from Fluc‐EGFP mice were pre‐incubated with 4‐PBA or vehicle control and then subjected to heat shock for 15 min at 43°C. In control slices, heat shock resulted in a marked reduction in Fluc‐EGFP‐specific luciferase activity. Importantly, 4‐PBA treatment led to a significant twofold increase in specific luciferase activity of heat‐shocked slices without a respective change in Fluc‐EGFP protein quantity (Fig 2C), indicating that Fluc‐EGFP response in brain tissue indeed reflects changes in proteostasis. In summary, we have generated a reporter mouse that can be used for monitoring neuronal proteostasis in the brain.

### Fluc‐EGFP reporter reveals proteostasis impairment in aging mice

Using Fluc‐EGFP mice, we first asked whether the reporter reacts to the *in vivo* changes in proteostasis capacity that are believed to occur in normal aging (Brehme *et al*, 2014; Labbadia & Morimoto, 2015; Klaips *et al*, 2018). To this end, we analyzed several brain regions of Fluc‐EGFP mice at different ages (3, 6, 12, and 24 months). We did not detect major changes in total Fluc‐EGFP protein levels between age groups (Fig 2D and F and Appendix Fig S2C). In 12‐ and 24‐month‐old mice, a high molecular weight smear was visible on the Western blot, likely representing aggregated Fluc‐EGFP species, and there was a corresponding reduction in the monomeric form (Fig 2D). Interestingly, we observed a significant age‐dependent decline in specific luciferase activity in the hippocampus and cortex, but not in the cerebellum (Fig 2E). These results show that Fluc‐EGFP mice can be used to monitor *in vivo* alterations in proteostasis and highlight intrinsic differences in the protein quality control of different brain regions.

### Fluc‐EGFP sensor reacts to proteostasis defects in tauopathy mice

**Figure 3:**
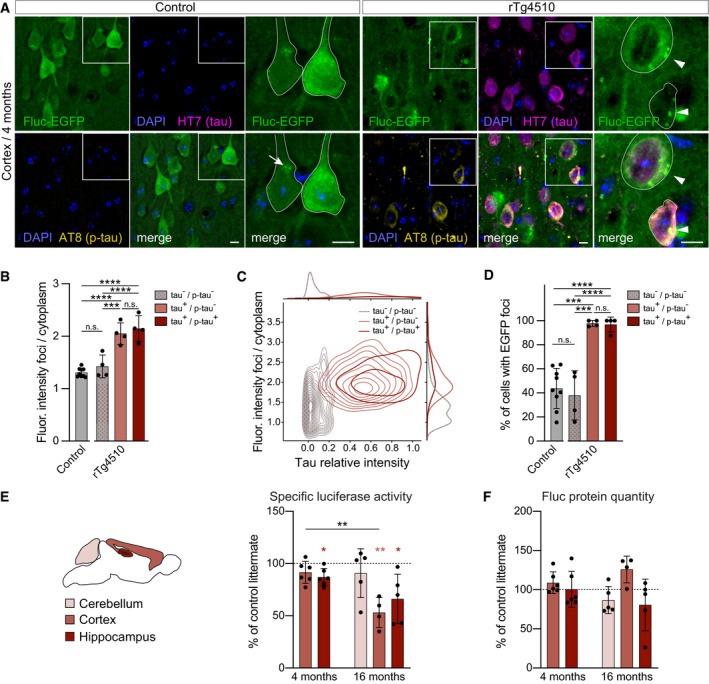
Fluc‐EGFP reporter reveals proteostasis impairment in tauopathy mice ACortical sections from rTg4510:Fluc‐EGFP mice (right) and control littermates (left) stained for total human tau (HT7, magenta) and p‐tau (AT8, yellow). Fluc‐EGFP was detected by EGFP fluorescence (green), and nuclei were labeled with DAPI (blue). Images on the right show higher magnification of the areas indicated by the boxes, with neuronal cell bodies outlined. Note the presence of EGFP foci (arrowheads) in the majority of neurons in rTg4510:Fluc‐EGFP mice. Occasional foci of lower relative fluorescence intensity can be observed in control mice (arrow).BQuantification of fluorescence intensity of EGFP foci, normalized to cytoplasmic EGFP fluorescence in cells with and without tau expression and p‐tau pathology. *N* = 4 rTg4510:Fluc‐EGFP mice, 3 WT:Fluc‐EGFP mice, 3 CaMKIIα‐tTA:Fluc‐EGFP mice, and 3 tetO‐tauP301L:Fluc‐EGFP mice. WT:Fluc‐EGFP, CaMKIIα‐tTA:Fluc‐EGFP, and tetO‐tauP301L:Fluc‐EGFP littermates did not differ from each other and were pooled together as controls. One‐way ANOVA with Tukey’s multiple comparisons test. ANOVA: ****P < 0.0001. Pairwise comparisons between groups are indicated on the graph.CContour plot of fluorescence intensity distribution of EGFP foci in cells with and without tau expression and p‐tau pathology. *N* = 174 tau^−^/p‐tau^−^ neurons; 178 tau^+^/p‐tau^−^ neurons, and 69 tau^+^/p‐tau^+^ neurons.DQuantification of the fraction of cells with EGFP foci among the cells with and without tau expression and p‐tau pathology. *N* = 4 rTg4510:Fluc‐EGFP mice, 3 WT:Fluc‐EGFP mice, 3 CaMKIIα‐tTA:Fluc‐EGFP mice, and 3 tetO‐tauP301L:Fluc‐EGFP mice. WT:Fluc‐EGFP, CaMKIIα‐tTA:Fluc‐EGFP, and tetO‐tauP301L:Fluc‐EGFP littermates did not differ from each other and were pooled together as controls. One‐way ANOVA with Tukey’s multiple comparisons test. ANOVA: ****P < 0.0001. Pairwise comparisons between groups are indicated on the graph.E, FLuciferase assay in rTg4510:Fluc‐EGFP mice. (E) Left, scheme of brain regions used for luciferase assay color‐coded by their vulnerability to tau pathology. Right, specific luciferase activity measured in the indicated brain regions of 4‐month‐old and 16‐month‐old rTg4510:Fluc‐EGFP mice normalized to control littermates. (F) Fluc‐EGFP protein quantity in the indicated brain regions of rTg4510:Fluc‐EGFP mice measured by Western blot (for representative blots, see Fig EV3B). Values are normalized to control littermates. *N* = 6 rTg4510:Fluc‐EGFP mice, 4 WT:Fluc‐EGFP mice, 6 CaMKIIα‐tTA:Fluc‐EGFP mice, and 1 tetO‐tauP301L:Fluc‐EGFP mouse at 4 months; 4–5 rTg4510:Fluc‐EGFP mice, 5 WT:Fluc‐EGFP mice, 3 CaMKIIα‐tTA:Fluc‐EGFP mice, and 4 tetO‐tauP301L:Fluc‐EGFP mice at 16 months. WT:Fluc‐EGFP, CaMKIIα‐tTA:Fluc‐EGFP, and tetO‐tauP301L:Fluc‐EGFP littermates did not differ from each other and were pooled together as controls. Light‐red and dark‐red asterisks indicate comparisons to the corresponding littermate group (one‐sample *t*‐test), and black asterisks indicate significant comparisons between corresponding brain regions of rTg4510:Fluc‐EGFP mice at 4 vs. 16 months (two‐tailed *t*‐test). Data information: Error bars represent SD. Significance: *P < 0.05; **P < 0.01; ***P < 0.001; ****P < 0.0001; n.s.—not significant. Scale bars in A, 10 µm.

**Figure EV3:**
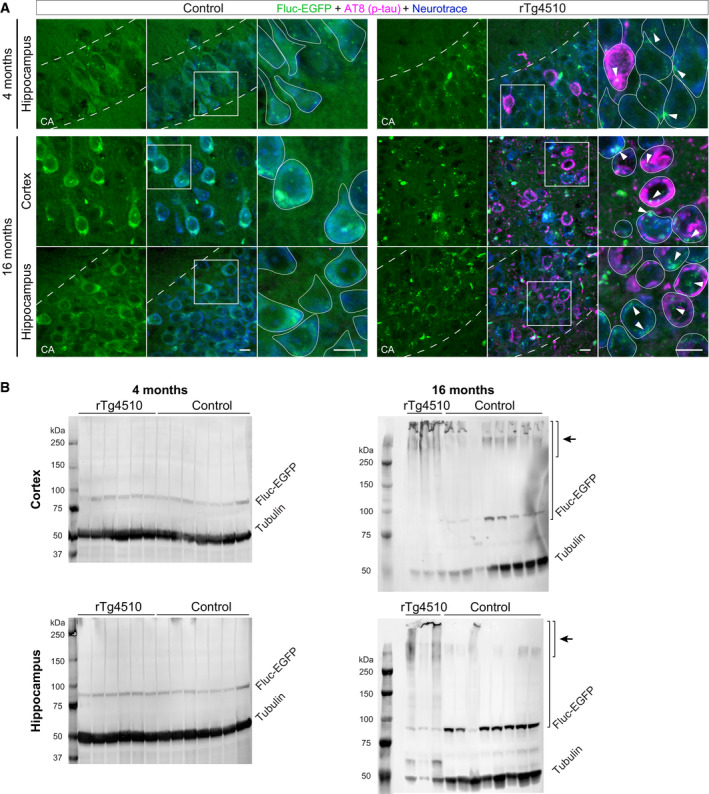
Additional analyses of Fluc‐EGFP reporter in rTg4510 mice Images of the cortex and hippocampus of control littermates (left) and rTg4510:Fluc‐EGFP mice (right) stained for p‐tau (AT8, magenta) at the indicated ages. Fluc‐EGFP was detected by EGFP fluorescence (green), neurons were labeled with Neurotrace (blue). Higher‐magnification images show the areas indicated by the boxes, with neuronal cell bodies outlined. Hippocampal CA layer is marked by dashed lines. Arrowheads point to Fluc‐EGFP foci. Note that there is little colocalization between EGFP foci and p‐tau.Representative Western blots of cortical (top) and hippocampal (bottom) lysates from 4‐month‐old (left) and 16‐month‐old (right) rTg4510:Fluc‐EGFP mice and control littermates. Short bracket with an arrow indicates the high molecular weight species observed in older mice. Long bracket indicates the part of the lane that was used for Fluc protein quantification. Data information: Scale bars in A, 10 µm. Source data are available online for this figure.

To investigate how proteostasis changes in disease, we crossed Fluc‐EGFP mice to the rTg4510 line, a double‐transgenic tauopathy model where expression of the responder transgene containing human four‐repeat tau with the familial P301L mutation (tetO‐tauP301L) is controlled by the CaMKIIα‐tTA activator transgene. rTg4510 mice develop neurofibrillary tangle‐like pathology and memory defects at 4 months, while neuronal loss and brain atrophy do not occur until 5.5 months of age (Santacruz *et al*, 2005). Consistent with previous reports (Santacruz *et al*, 2005; Myeku *et al*, 2016), immunostaining for phosphorylated tau (p‐tau) revealed tau pathology in the cortex and hippocampus of 4‐month‐old rTg4510:Fluc‐EGFP mice (Figs 3A and Fig EV3A). At this time point, multiple Fluc‐EGFP foci were observed in both cortex and hippocampus. While foci were also seen in control mice, they were less frequent, and their relative fluorescence intensity was clearly lower than in rTg4510 mutants (Figs 3A and Fig EV3A).

Interestingly, co‐staining for total human tau and p‐tau showed that the relative intensity of Fluc‐EGFP foci, as well as the fraction of cells with foci, were similarly increased in all human tau‐positive cells of rTg4510 mice, regardless of the presence of p‐tau pathology (Fig 3A–D). In contrast, in human tau‐negative cells of rTg4510 mice the occurrence and relative intensity of foci were not different from control mice (Fig 3B and D). These results suggest that Fluc‐EGFP is sensitive to protein aggregation in the cytoplasm prior to formation of insoluble aggregates. Moreover, there was very little colocalization between p‐tau and Fluc‐EGFP foci in p‐tau‐positive cells (Figs 3A and Fig EV3A), arguing that Fluc aggregation was truly due to impaired proteostasis rather than trapping of the sensor within the tau aggregates.

The luciferase assay revealed a small, but significant decrease in specific Fluc enzymatic activity in the hippocampus, but not cortex of 4‐month‐old rTg4510 mice (Fig 3E). The modest change in luciferase activity in total tissue lysates might be due to the variability of cellular reactions to misfolding *in vivo* at this early stage of pathology that might mask proteostasis impairments occurring in a fraction of cells. We therefore examined advanced‐stage 16‐month‐old rTg4510 mice that show very abundant tau neurofibrillary tangles (Fig EV3A). At this age, a clear decrease in specific luciferase activity was detected both in the cortex and hippocampus, but not in the cerebellum, where the tau transgene is not expressed (Fig 3E). Total Fluc‐EGFP protein quantity was not significantly altered in any of the brain regions examined (Fig 3F). However, high molecular weight species of Fluc‐EGFP that were observed in all aged mice appeared more prominent in 16‐month‐old rTg4510 mutants, while the levels of Fluc‐EGFP monomers were decreased compared to controls (Fig EV3B). These results suggest that the solubility of the Fluc‐EGFP sensor decreases with the progression of tau pathology. Taken together, our findings in tauopathy mice demonstrate the ability of Fluc‐EGFP to react to proteostasis defects caused by expression of an aggregating protein *in vivo*.

### Fluc‐EGFP sensor does not detect proteostasis changes in HD mice

**Figure 4:**
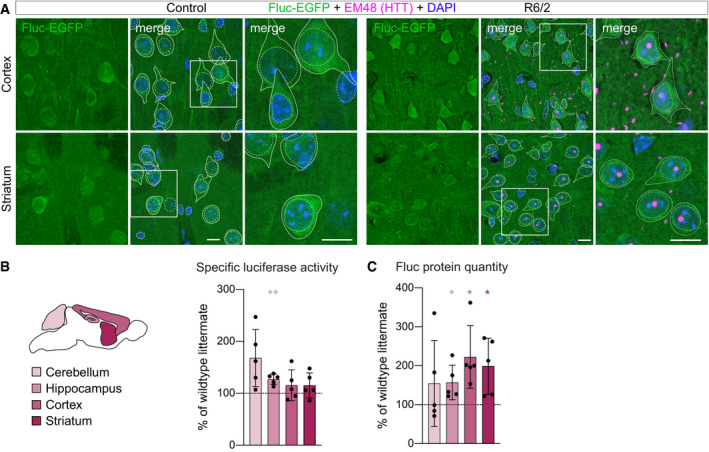
Fluc‐EGFP reporter does not detect proteostasis impairment in R6/2 mice ACortical (upper row) and striatal (lower row) sections from 12‐week‐old R6/2:Fluc‐EGFP mice (right) and control WT:Fluc‐EGFP littermates (left) stained for aggregated mHTT (EM48, magenta). Fluc‐EGFP was detected by EGFP fluorescence (green), and nuclei were counterstained with DAPI (blue). Images on the right show higher magnification of the areas indicated by the boxes. Continuous and stippled lines mark cell bodies and nuclei, respectively.B, CLuciferase assay in R6/2 mice. (B) Left, scheme of brain regions used for luciferase assay color‐coded by their vulnerability to Huntington’s disease. Right, specific luciferase activity measured in the indicated brain regions of 12‐week‐old R6/2:Fluc‐EGFP mice normalized to WT:Fluc‐EGFP littermates. (C) Fluc‐EGFP protein quantity in the indicated brain regions of R6/2:Fluc‐EGFP mice measured by Western blot. Values are normalized to WT:Fluc‐EGFP littermates. *N* = 5 mice of each genotype. Colored asterisks in B and C indicate significant comparisons to the respective brain regions of control littermates (one‐sample *t*‐test). Data information: Error bars represent SD. Significance: *P < 0.05; **P < 0.01. Scale bars in A, 10 µm.

In addition to tauopathy mice, we investigated proteostasis in the R6/2 mouse model of HD. R6/2 is an early‐onset transgenic model that expresses mHTT‐exon1 under the human HTT promoter (Mangiarini *et al*, 1996). The mice have a rapid disease progression, showing frequent mHTT inclusion bodies (mHTT IBs) at the age of 3 weeks, and severe brain shrinkage along with marked motor impairments at 12 weeks (Mangiarini *et al*, 1996; Carter *et al*, 1999; Meade *et al*, 2002; Burgold *et al*, 2019). In contrast to rTg4510 mice, we did not observe any changes in cellular distribution of Fluc‐EGFP in R6/2:Fluc‐EGFP mice, even at the late symptomatic stage of 12 weeks when mHTT IBs were present in every cell (Fig 4A). Consistently, no decrease in specific luciferase activity was observed in the cerebellum, hippocampus, cortex, or striatum of 12‐week‐old R6/2:Fluc‐EGFP mutants compared to littermate WT:Fluc‐EGFP controls (Fig 4B), although measurements of protein quantity revealed a significant increase in Fluc‐EGFP levels in the hippocampus, cortex, and striatum of R6/2 mice (Fig 4C). These results are compatible with a scenario where Fluc degradation might be impaired, but Fluc is nevertheless properly folded in the R6/2 brain.

**Figure EV4:**
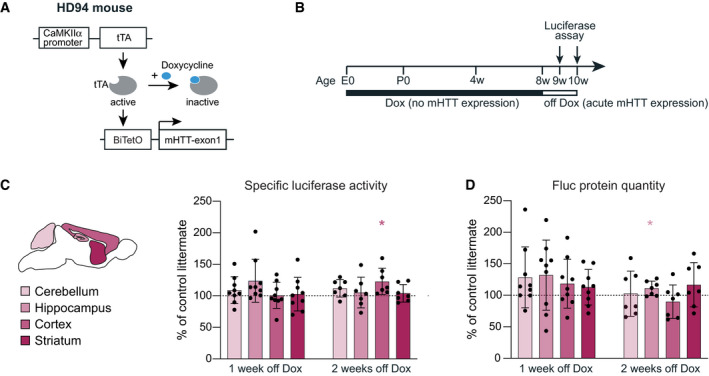
Proteostasis measurements in HD94:Fluc‐EGFP mice upon acute induction of mHTT expression AScheme of the HD94 transgenic strategy.BExperimental timeline. E0, embryonic day 0; P0, postnatal day 0.C, DLuciferase assay in HD94 mice. (C) Left, scheme of brain regions used for luciferase assay color‐coded by their vulnerability to Huntington’s disease. Right, specific luciferase activity of cerebellar, hippocampal, cortical, and striatal tissue lysates. (D) Fluc‐EGFP protein levels measured by Western blot. WT:Fluc‐EGFP, CaMKIIα‐tTA:Fluc‐EGFP, and BiTetO:Fluc‐EGFP littermates did not differ from each other and were pooled together as controls. Values of HD94:Fluc‐EGFP mice were normalized to their respective pooled littermate controls. 1 week off Dox: *N* = 9 HD94:Fluc‐EGFP mice; 5 CaMKIIα‐tTA:Fluc‐EGFP mice; 7 BiTetO:Fluc‐EGFP mice; and 7 WT:Fluc‐EGFP mice; 2 weeks off Dox: 7 HD94:Fluc‐EGFP mice; 2 CaMKIIα‐tTA:Fluc‐EGFP mice; 3 BiTetO:Fluc‐EGFP mice; and 3 WT:Fluc‐EGFP mice. Colored asterisks in C and D indicate significant comparisons to the respective brain regions of control littermates (one‐sample *t*‐test). Data information: Error bars represent SD. Significance: *P < 0.05.

Previous studies with UPS reporters also failed to show UPS impairments in HD mouse models with constitutive expression of mHTT (Bett *et al*, 2009; Maynard *et al*, 2009; Ortega *et al*, 2010). However, a transient defect in UPS was detected in the inducible HD94 model after mHTT expression was acutely switched on (Ortega *et al*, 2010). These previous observations raised the possibility that mHTT may initially impose a burden on the proteostasis system, which in the long term might be overcome by compensatory mechanisms. To test this, we crossed Fluc‐EGFP mice to the inducible HD94 mouse line (CaMKIIα‐tTA:BiTetO‐HTT‐Q94) (Yamamoto *et al*, 2000), which allows for precise temporal control over mHTT‐exon1 expression (Fig EV4A). The breeding pairs and the offspring were kept on Doxycycline throughout pregnancy and postnatally to suppress mHTT and prevent any compensatory adaptations (Fig EV4B). Doxycycline treatment was stopped at 8 weeks of age, and brains were harvested one and two weeks later, time points when expression of the mHTT transgene is already detectable and the most prominent UPS impairment occurs (Ortega *et al*, 2010). As mHTT is only expressed in the forebrain in the HD94 model, the cerebellum served as a negative control in these experiments. In agreement with our findings in the R6/2 line, no decrease in luciferase activity was observed in the hippocampus, cortex, or striatum of HD94:Fluc‐EGFP mice 1 week or 2 weeks after mHTT transgene induction (Fig EV4C). We did not detect any differences in Fluc‐EGFP protein quantity in this model compared to littermate controls (Fig EV4D). These data suggest that also acute expression of mHTT is not sufficient to cause proteostasis defects. Taken together, our findings indicate that the Fluc‐EGFP sensor does not detect impairments of protein quality control in two different HD models.

### Cellular compartment‐specific reactions of Fluc‐EGFP to different aggregating proteins

**Figure 5:**
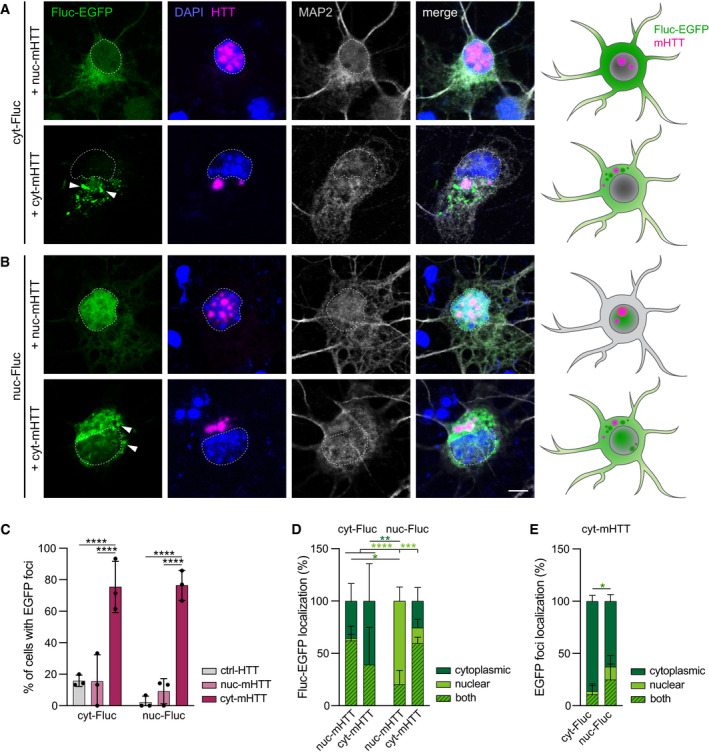
Response of nuclear and cytoplasmic Fluc‐EGFP to nuclear and cytoplasmic mHTT A, BPrimary cortical neurons transfected with cyt‐Fluc‐EGFP (A) or nuc‐Fluc‐EGFP (B) (green), in combination with nuc‐mHTT (upper rows) or cyt‐mHTT (lower rows) (magenta). Cells were fixed at DIV 3 + 2 and stained for the neuronal marker MAP2 (gray). Nuclei were labeled with DAPI (blue). Arrowheads point to Fluc‐EGFP foci. Dashed lines mark the nuclei. Schemes on the right summarize the cellular distribution of the respective Fluc‐EGFP (green) and mHTT (magenta) proteins. Corresponding cultures transfected with control HTT are shown in Appendix Fig S3A.CQuantification of Fluc‐EGFP foci formation. *N* = 3 independent experiments. Two‐way ANOVA with Tukey’s multiple comparisons test. ANOVA: HTT, ****P < 0.0001; Fluc, n.s.; Interaction HTT × Fluc, n.s. Significant pairwise comparisons within the respective Fluc‐EGFP group are indicated above the bars.DQuantification of the subcellular localization of the respective Fluc‐EGFP protein in the presence of the indicated mHTT constructs. *N* = 3 independent experiments. Three‐way ANOVA with Tukey’s multiple comparisons test. ANOVA: Localization, *P = 0.019; HTT, n.s.; Fluc, n.s.; Localization x HTT, **P = 0.0012; Localization × Fluc, ****P < 0.0001; HTT × Fluc, n.s.; Localization × HTT × Fluc, ***P = 0.0009.EQuantification of Fluc‐EGFP foci localization in the presence of cyt‐mHTT. *N* = 3 independent experiments. Two‐way ANOVA with Bonferroni’s multiple comparisons test. ANOVA: Localization, ****P < 0.0001; Fluc, n.s.; Localization × Fluc, *P = 0.01. Dark‐green, light‐green and striped asterisks in D and E indicate significant comparisons of the fractions of cells with Fluc‐EGFP localized in the cytoplasm, in the nucleus, or in both compartments, respectively. Data information: Error bars represent SD. Significance: *P < 0.05; **P < 0.01; ***P < 0.001; ****P < 0.0001. Scale bar in A, B, 5 µm.

One difference between the disease models studied here is that in rTg4510 mice, aggregated tau is localized exclusively in the cytoplasm, including prominent pathology in the cell body, whereas both HD models display abundant mHTT IBs in the nucleus, but not in the cell body (Figs 3A and Fig EV3A, and Fig 4A; Davies *et al*, 1997; Santacruz *et al*, 2005; Yamamoto *et al*, 2000). We therefore tested whether the Fluc‐EGFP sensor is able to detect mHTT‐dependent nuclear proteostasis impairments when targeted to the nucleus. To this end, we co‐expressed FlucWT‐EGFP versions targeted to the nucleus (NLS‐Fluc‐EGFP, from here on nuc‐Fluc‐EGFP) and to the cytoplasm (NES‐Fluc‐EGFP, from here on cyt‐Fluc‐EGFP) together with mCherry‐fused versions of mHTT‐exon1 that localize predominantly to the nucleus (mCherry‐HTT‐Q74, from here on nuc‐mHTT) (Pan *et al*, 2018) or to the cytoplasm (HTT‐Q97‐mCherry, from here on cyt‐mHTT) (Hipp *et al*, 2012). Both nuc‐mHTT and cyt‐mHTT readily aggregated and formed IBs in the respective compartment (Fig 5A and B and Appendix Fig S3B). Remarkably, cyt‐mHTT caused proteostasis impairments that were detected by both cytoplasmic‐ and nuclear‐targeted Fluc‐EGFP (Fig 5A–C). Conversely, nuc‐mHTT did not cause foci formation neither by cyt‐Fluc‐EGFP, nor by nuc‐Fluc‐EGFP (Fig 5A–C). Moreover, in the presence of cyt‐mHTT, we often observed mislocalization of nuc‐Fluc‐EGFP to the cytoplasm and appearance of cytoplasmic Fluc‐EGFP foci, which were more abundant than nuclear ones (Fig 5B,D,E). Similar to our observations with p‐tau in rTg4510 mice, no colocalization was detected between cyt‐mHTT IBs and Fluc‐EGFP foci (Fig 5A and B). In summary, the Fluc‐EGFP sensor reacts to the presence of cytoplasmic, but not nuclear, mHTT aggregates, suggesting that cytoplasmic mHTT aggregates might cause a greater disturbance of neuronal proteostasis.

**Figure EV5:**
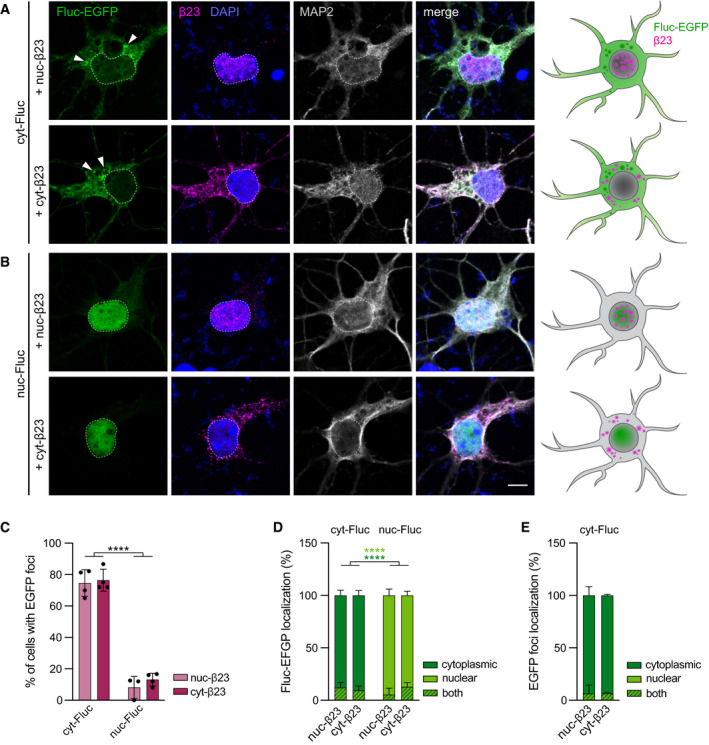
Response of nuclear and cytoplasmic Fluc‐EGFP to nuclear and cytoplasmic β23 protein A, BPrimary cortical neurons transfected with cyt‐Fluc‐EGFP (A) or nuc‐Fluc‐EGFP (B) (green), in combination with nuc‐β23 (upper rows) or cyt‐β23 (lower rows). Cells were fixed at DIV 3 + 2 and stained for myc to detect β23 (magenta) and for the neuronal marker MAP2 (gray). Nuclei were labeled with DAPI (blue). Arrowheads point to Fluc‐EGFP foci. Dashed lines mark the nuclei. Schemes on the right summarize the cellular distribution of the respective versions of Fluc‐EGFP (green) and β23 (magenta).CQuantification of Fluc‐EGFP foci formation. *N* = 3–4 independent experiments. Two‐way ANOVA with Tukey’s multiple comparisons test. ANOVA: β23, n.s.; Fluc, ****P < 0.0001; β23 × Fluc, n.s. Significant pairwise comparisons are indicated on the graph.DQuantification of the subcellular localization of the respective Fluc‐EGFP protein in the presence of the indicated β23 versions. *N* = 3–4 independent experiments. Three‐way ANOVA with Tukey’s multiple comparisons test. ANOVA: Localization, ****P < 0.0001; β23, n.s., Fluc, n.s.; Localization × β23, n.s.; Localization × Fluc, ****P < 0.0001; β23 × Fluc, n.s.; Localization × β23 × Fluc, *P = 0.0135. Dark‐green and light‐green asterisks indicate significant pairwise comparisons of the fractions of cells with Fluc‐EGFP localized in the cytoplasm or in the nucleus, respectively.EQuantification of cyt‐Fluc‐EGFP foci localization in the presence of nuc‐β23 or cyt‐β23. *N* = 3–4 independent experiments. Two‐way ANOVA with Bonferroni’s multiple comparisons test. ANOVA: Localization, ****P < 0.0001; β23, n.s.; Localization × β23, n.s. Data information: Error bars represent SD. Significance: ****P < 0.0001. Scale bar in A, B, 5 µm.

To test whether this observation holds true for other aggregating proteins, we performed similar experiments with the rationally designed aggregating protein β23 (West *et al*, 1999; Olzscha *et al*, 2011), which has previously been used to explore the properties of protein aggregates in different cellular compartments (Woerner *et al*, 2016; Vincenz‐Donnelly *et al*, 2018). Nuclear and cytoplasmic β23 (NLS‐myc‐β23 and myc‐NES‐β23, from here on nuc‐β23 and cyt‐β23, respectively) predominantly localized to and formed aggregates in the respective cellular compartment (Fig EV5A and B and Appendix Fig S3C). In contrast to our findings with mHTT, in these experiments we observed very similar reaction of the Fluc sensor versions to nuclear vs. cytoplasmic aggregates: While cyt‐Fluc‐EGFP formed abundant foci in response to both nuc‐β23 and cyt‐β23, nuc‐Fluc‐EGFP only formed very few foci in either condition (Fig EV5A–E). This is compatible with a scenario where β23, regardless of its localization, disturbs cytoplasmic, but not nuclear proteostasis. Taken together, these results suggest that different aggregating proteins cause distinct compartment‐specific proteostasis impairments.

## Discussion

Here, we have generated new transgenic mice with expression of a proteostasis reporter in the brain and demonstrated their utility in monitoring proteostasis alterations in aging and disease. Proteostasis defects in the brain can be detected both by Fluc‐EGFP foci formation, as well as by decline in luciferase activity. In contrast to available UPS reporters (Lindsten *et al*, 2003; Bove *et al*, 2006) that are limited to the analysis of protein degradation, Fluc‐EGFP mice allow assessing the entire protein quality control system of neurons. It should be kept in mind that the expression of an unstable protein such as Fluc might itself impose a burden on the cellular quality control machinery and lead to long‐term changes in the proteostasis network. In our transgenic line, the sensor is expressed at a mild level, mitigating potential adaptive changes. In addition, our experiments with brain slices (Fig 2C), along with previous investigations in HeLa cells (Gupta *et al*, 2011), showed that Fluc‐EGFP only has a minor effect on proteostasis and does not alter cellular stress responses. Using the sensor in the context of inducible genetic models and viral‐based strategies is an exciting possibility for future studies that would further minimize this limitation.

We used the Fluc‐EGFP mice to probe proteostasis alterations in models of two different neurodegenerative proteinopathies caused by expression of mutant tau and mHTT. In tauopathy mice, the sensor revealed an impairment of proteostasis already at an early stage of disease. Of note, in our experiments with 4‐month‐old rTg4510 animals, impaired proteostasis could be detected more readily by changes in Fluc‐EGFP cellular distribution than by changes in specific luciferase activity (Fig 3). This is in contrast to cell culture conditions where the luciferase activity readout showed a higher sensitivity to proteotoxic stress (Gupta *et al*, 2011). A possible reason for this difference might be the heterogeneity of cell types in the brain which differ in their proteostasis machinery and their responses to misfolding. These observations suggest that early changes in proteostasis might be difficult to detect with a luciferase activity‐based assay in bulk tissue and emphasize the importance of single‐cell resolution approaches when studying protein misfolding in complex tissues *in vivo*. Combined with recent developments in single‐cell RNA sequencing techniques, our reporter mouse offers an experimental tool to uncover the molecular basis of proteostasis differences between various cell types, by comparing the transcriptional signatures of cells with proteostasis differences revealed by the reporter.

In contrast to tauopathy mice, the Fluc‐EGFP sensor did not show any reaction in HD mice. As these disease models are based on different genetic strategies and have different overexpression levels of the respective pathogenic protein, comparisons between them should be made with caution. Nevertheless, our findings are in line with previous studies using UPS reporters, which demonstrated UPS impairment in the rTg4510, but not R6/2 model (Bett *et al*, 2009; Maynard *et al*, 2009; Myeku *et al*, 2016). As there is extensive evidence of UPS defects in HD, the negative results obtained with UPS sensors and with our sensor could be partially due to the long‐term compensatory changes in mice with constitutive expression of mHTT (Ortega *et al*, 2010; Ortega & Lucas, 2014). However, the observations we made in the HD94 line are seemingly in contrast to a previous report, where accumulation of the UPS reporter Ub‐G76V‐GFP was detected upon acute induction of mHTT expression (Ortega *et al*, 2010). Of note, our results do not exclude the possibility that protein degradation by UPS might be impaired in HD94 mice, however, they suggest that other components of the protein quality control machinery may compensate for the UPS defect.

Our mechanistic investigations in cultured neurons suggest that both the nature of the aggregates and their subcellular localization might contribute to the differences in proteostasis between disease models. We observed that β23 aggregates had an effect on the solubility of cytoplasmic Fluc‐EGFP, regardless of where the aggregates themselves were localized, while mHTT‐exon1 IBs only induced Fluc reaction when they were localized in the cytoplasm. Although mHTT is also found in the cytoplasm of HD mouse models used here (Yamamoto *et al*, 2000; Osmand *et al*, 2016; Landles *et al*, 2020), it does not seem to have a strong impact on the proteostasis network, in contrast to the effects of cytoplasmic mHTT IBs that we and others observed in cultured cells (Gupta *et al*, 2011). These differences might be caused by the localization of cytoplasmic IBs predominantly in neurites, but not in the cell body of neurons in HD mice, by higher expression levels of mHTT in cellular models compared to transgenic mice, and/or by the shorter duration of mHTT expression in cell culture, which provides less room for compensatory adaptations.

While we cannot rule out differences in the sensitivity of the Fluc‐EGFP sensor to proteostasis impairments in different cellular compartments, our results support the idea that nuclear and cytoplasmic compartments differ in their capacity to cope with protein aggregation. This is in agreement with studies in yeast and in cell lines describing distinct protein quality control mechanisms operating in the nucleus and cytoplasm (Hageman *et al*, 2007; Park *et al*, 2013; Woerner *et al*, 2016; Enam *et al*, 2018; Samant *et al*, 2018; Frottin *et al*, 2019; den Brave *et al*, 2020; Raeburn *et al*, 2021). Although we did not observe any Fluc‐EGFP response to mHTT IBs localized in the nucleus, it should be noted that our findings do not exclude deleterious effects of nuclear mHTT, which have been demonstrated previously in cultured cells and animal models (Saudou *et al*, 1998; Yang *et al*, 2002; Bae *et al*, 2006; Gu *et al*, 2015; Veldman *et al*, 2015). Instead, they are compatible with the notion that nuclear and cytoplasmic mHTT aggregates have different molecular properties and are involved in different aspects of the disease (Landles *et al*, 2020). Our data suggest that toxicity of nuclear mHTT is likely due to other mechanisms than impaired proteostasis and therefore might not be amenable to treatments targeting the proteostasis network. This provides a possible explanation for the weak effects of chaperone overexpression in mouse HD models that show prominent nuclear mHTT pathology (Hansson *et al*, 2003; Zourlidou *et al*, 2007; Labbadia *et al*, 2012), while similar strategies proved successful in rescuing toxicity caused by cytoplasmic polyQ fragments in cell lines and in flies (Gunawardena *et al*, 2003; Hageman *et al*, 2010; Jiang *et al*, 2012).

An unexpected finding in cyt‐mHTT cells was the mislocalization of nuc‐Fluc‐EGFP to the cytoplasm (Fig 5B and D). We speculate that it might be due to dysregulated nucleocytoplasmic transport that can occur in the presence of cytoplasmic aggregating proteins (Woerner *et al*, 2016; Li & Lagier‐Tourenne, 2018), including mHTT (Gasset‐Rosa *et al*, 2017; Grima *et al*, 2017).

In summary, our new proteostasis reporter mice represent a useful tool for detailed studies of proteostasis in various proteinopathy models and can be used in the future for monitoring the success of therapeutic interventions that target the cellular protein quality control system. With the help of these mice, we uncovered unexpected differences in the impact of different aggregating proteins on the cellular protein quality control system. Our results therefore suggest that the therapeutic success of enhancing the proteostasis system in different diseases might depend on the nature of the aggregates. In future studies, it will be interesting to use the Fluc‐EGFP reporter mice in the context of other protein misfolding diseases beyond tauopathy and HD.

## Materials and Methods

### Expression constructs

The following plasmids were used in the study: FlucWT‐EGFP and FlucSM‐EGFP (Gupta *et al*, 2011) with myc tag exchanged for HA; NLS‐Fluc‐EGFP and NES‐Fluc‐EGFP (Park *et al*, 2013); Mo.PrP (Borchelt *et al*, 1996); myc‐NES‐β23 and NLS‐myc‐β23 (Woerner *et al*, 2016); HTT‐Q25‐mCherry and HTT‐Q97‐mCherry (Hipp *et al*, 2012); and EGFP‐HTT‐Q74 (Addgene 40262) with EGFP exchanged for mCherry. The sequences of the nuclear (mCherry‐HTT‐Q74) and cytoplasmic (HTT‐Q97‐mCherry, HTT‐Q25‐mCherry) HTT‐exon1 constructs are similar apart from the polyQ length and the 7 N‐terminal amino acids and 33 C‐terminal amino acids of HTT‐exon 1, which are present in the cytoplasmic, but not in the nuclear constructs.

### Mice

All the animal experiments were approved by the Government of Upper Bavaria (animal protocols 55.2‐1‐54‐2532‐13‐13 and 55.2‐1‐54‐2532‐168‐14) and conducted in accordance with the ARRIVE guidelines and relevant regulations. Mice were maintained in a specific pathogen‐free animal facility with *ad libitum* access to food and water. Animals of either sex were used for experiments, and no specific randomization or blinding was performed.

For generation of Fluc‐EGFP transgenic lines, FlucWT‐EGFP and FlucSM‐EGFP sequences with a C‐terminal HA tag were cloned into the Mo.PrP plasmid kindly provided by David Borchelt (University of Florida). Transgenic mice were generated by microinjection of linearized transgenic constructs into the pronucleus of C57BL/6 oocytes. Integration of the transgene was detected by PCR with the following primers: forward, 5’‐GTG TCG CTC TGC CTC ATA GAA CTG CCT GCG TG‐3’; reverse, 5’‐CAT CCT TGT CAA TCA AGG CGT TGG TCG CTT CCG‐3’. Fluc‐EGFP mice were kept on C57BL/6 background.

R6/2 mice (Mangiarini *et al*, 1996) were obtained from JaxLabs (Stock No. 002810) and maintained by crossing transgenic males to F1 CBA/BL6 wild‐type females. CAG repeat length was determined from tail biopsies by Laragen, Inc, and amounted to 192 ± 2 repeats. To obtain HD94 mice (Yamamoto *et al*, 2000), BiTetO mice (kind gift of José J. Lucas, Universidad Autonoma de Madrid) were crossed to the CaMKIIα‐tTA line (Mayford *et al*, 1996) (JaxLabs, Stock No. 003010). HD94 breeding pairs and offspring were treated with 2 mg/ml doxycycline, 5% sucrose in drinking water from conception until 2 months of age. rTg4510 mice (Santacruz *et al*, 2005) were obtained by crossing tetO‐tauP301L mice (JaxLabs, Stock No. 015815) to the CaMKIIα‐tTA line and maintained on an FVB genetic background.

### Primary neuronal cultures

Sterile 13 mm glass coverslips were coated with 0.5 mg/ml poly‐D‐lysine overnight at 37°C and with 5 µg/ml Laminin in PBS for 2–4 h at 37°C. Primary cortical neurons were prepared from E15.5 CD1 embryos. Pregnant females were sacrificed by cervical dislocation, the uterus was removed from the abdominal cavity, embryos were harvested and decapitated in ice‐cold dissection medium consisting of Hanks’ balanced salt solution (HBSS) supplemented with 0.01 M HEPES, pH 7.4, 0.01 M MgSO_4_, and 1% Penicillin/Streptomycin. The skull was cut open, and the cerebral hemispheres were separated from the rest of the brain. After removing the meninges, cortices were dissected and digested with pre‐warmed 0.25% Trypsin‐EDTA supplemented with 0.75% DNAse for 15 min at 37°C. Trypsin activity was quenched by washing with Neurobasal medium containing 5% fetal bovine serum (FBS), and cells were dissociated in pre‐warmed culture medium by triturating. Cells were centrifuged at 130 *g* for 5 min and the pellet was resuspended in culture medium consisting of Neurobasal medium with 2% B27 (Invitrogen), 1% l‐glutamine (Invitrogen), and 1% penicillin/streptomycin (Invitrogen). Cells were plated in 24‐well plates at a density of 100,000 per well. Transfection was performed at DIV 3 using CalPhos Mammalian Transfection Kit (TakaRa/Clontech). Briefly, coverslips were transferred into a new 24‐well plate with fresh pre‐warmed culture medium. Cells were incubated with 30 µl of the transfection mix (1.5 µg DNA, 124 mM CaCl_2_, 1× HBS in H_2_O) at 37°C, 5% CO_2_ for 3 h. A 24‐well plate with fresh culture medium was equilibrated for at least 30 min in 10% CO_2_, and the coverslips were transferred into the equilibrated plate for 30 min at 37°C, 5% CO_2_. Finally, coverslips were moved back to the original medium and incubated at 37°C, 5% CO_2_ for protein expression. 4‐PBA (Sigma, SML0309) was dissolved in H_2_O, and cells were treated with 1 mM 4‐PBA or H_2_O as control. MG‐132, Bafilomycin A1, and 17‐AAG were diluted in 0.1% DMSO, and respective control samples were treated with 0.1% DMSO alone.

### Acute brain slices

350 µm thick acute brain slices were prepared on a vibratome (Leica VT1000S) in ice‐cold cutting solution (30 mM NaCl, 4.5 mM KCl, 1 mM MgCl_2_, 26 mM NaHCO_3_, 1.2 mM NaH_2_PO_4_, 10 mM glucose, and 194 mM sucrose, saturated with a mixture of 95% O_2_ and 5% CO_2_). Slices were equilibrated at 30°C for 30 min in artificial CSF (aCSF) solution (124 mM NaCl, 4.5 mM KCl, 1 mM MgCl_2_, 26 mM NaHCO_3_, 1.2 mM NaH_2_PO_4_, 10 mM glucose, and 2 mM CaCl_2_ (310–320 mOsm), saturated with a mixture of 95% O_2_ and 5% CO_2_) before being incubated at 37°C in aCSF supplied with the indicated compounds (5 µM MG‐132 for 4 h; 1 mM 4‐PBA for 1 h, followed by 15 min heat shock at 43°C).

### Immunofluorescence

Mice were deeply anesthetized with 1.6% Ketamine/0.08% Xylazine and transcardially perfused with PBS followed by 4% paraformaldehyde (PFA) in PBS. Brains were dissected out of the skull and post‐fixed in 4% PFA in PBS overnight. Fixed tissue was embedded in agarose and sectioned with a vibratome (VT1000S, Leica). Sections were rehydrated in PBS and permeabilized with 0.5% Triton X‐100. After washing with PBS, cells were incubated in blocking solution consisting of 0.2% bovine serum albumin (BSA), 5% donkey serum, 0.2% lysine, 0.2% glycine in PBS for 30–60 min at room temperature (RT). Primary antibodies were applied in 0.3% Triton X‐100, 2% BSA in PBS overnight at 4°C. The following primary antibodies were used: GFP (Life technologies, A11122), NeuN (Millipore, MAB377), HT7 (Invitrogen, MN1000), AT8 (Invitrogen, MN1020), and EM48 (Millipore, MAB5374). Double stainings with AT8 and HT7 were performed sequentially, with an intermediate blocking step (5% mouse serum). After washing three times with PBS, fluorescently labeled secondary antibodies and Neurotrace™ 435 / 455 (Thermo Fisher, N21479, 1:200) were applied in 0.3% Triton X‐100, 3% donkey serum for 1–2 h at RT. DAPI was added at 1:1,000 dilution in PBS. Before mounting, the sections were treated with 0.5% Sudan Black B solution in 70% EtOH for 1 min in order to quench autofluorescence. Sections were mounted with fluorescent mounting medium (DAKO) or ProLong Glass Antifade medium (Invitrogen).

Cultured neurons were fixed with 4% PFA in PBS, washed with PBS, and permeabilized with 0.1% Triton X‐100 in PBS for 10 min at RT. After washing with PBS, cells were incubated in blocking solution (2% bovine serum albumin, 4% donkey serum in PBS) for 30 min at RT. Primary antibodies were applied in blocking solution for 1 h at RT. The following primary antibodies were used: cleaved caspase‐3 (Abcam, ab13847), MAP‐2 (Novus, NB300‐213), and myc (CST, 2272). After washing three times with PBS, fluorescently labeled secondary antibodies were applied in blocking solution for 30 min at RT. DAPI was added at 1:1,000 dilution in PBS. Coverslips were mounted with fluorescent mounting medium (DAKO).

All images were acquired using a Leica TCS SP8 scanning confocal microscope. Images were processed and analyzed with Fiji‐ImageJ. Quantification of localization and aggregation of Fluc‐EGFP, HTT‐exon1, and β23 in primary neurons was performed manually. Wherever possible, the investigator was blinded to the treatment of primary neurons in order to assess the reaction of the Fluc‐EGFP sensor. To determine the fraction of Fluc‐EGFP foci‐containing cells in brain sections, a threshold of 1.5 standard deviations above Fluc‐EGFP foci/cytoplasm intensity ratio was defined as decision boundary for the presence of foci.

### Luciferase assay and Western blotting

Mice were sacrificed by cervical dislocation, brains were quickly removed from the skull, and brain regions were dissected on ice. Brain region samples and brain slices were homogenized in pre‐chilled lysis buffer (50 mM Tris–HCl, pH 7.4, 150 mM NaCl, 1% Triton X‐100, protease inhibitor cocktail (Roche, 04693132001) and phosphatase inhibitor (Roche, 04906837001)). Primary neurons were harvested in the same lysis buffer. Lysates were centrifuged for 15 min at 14,000 *g* and 4°C, and supernatants were collected and used for the luciferase assay and Western blot. For luciferase assay, samples containing 100 µg total protein were mixed with 100 µl Luciferase Substrate (Promega). The kinetics of the luminescence was recorded for 10 min using a TriStar² S LB 942 Plate Reader (Berthold Technologies), and the maxima of the curves were extracted. For Western blot, tissue samples containing 100 µg total protein were denatured, separated on a 4‐15% gradient gel (Criterion™ TGX Stain‐Free™Precast Gel, Bio‐Rad), activated using a ChemiDoc Reader (Bio‐Rad), and transferred onto a PVDF membrane using a Trans‐Blot Turbo transfer system (Bio‐Rad). Neuronal lysates from primary cultures containing 20 µg total protein were loaded on a 10% gel (Mini‐PROTEAN**^®^** TGX Stain‐Free™ Precast Gel, Bio‐Rad) for Akt detection and a 4‐20% gradient gel for ubiquitin and LC3B detection. After blocking with 5% milk, 3% BSA, primary antibodies in 3% BSA, 0.1% Tris‐buffered saline with 0.1% TWEEN® 20 (TBS‐T) were incubated overnight at 4°C, followed by secondary antibodies in 3% milk, 0.1% TBS‐T for 1 h at RT. The following primary antibodies were used: GFP (JL‐8, Clontech, 632381), tubulin (Covance, MMS‐435P), ubiquitin (CST, 3936), LC3B (CST, 43566), Akt (CST, 2920), and p‐Akt (CST, 4060). Proteins were visualized by fluorescence using the ChemiDoc Reader (Bio‐Rad). To take potential high‐molecular weight smear of Fluc signal into account, the entire background‐adjusted lane area above the Fluc‐EGFP monomer band was quantified using Image Lab™ Software (Bio‐Rad) (see Figs 2D and Fig EV3B). Total protein amount was determined using stain‐free technology (Bio‐Rad) which relies on in‐gel chemistry and visualizes proteins containing tryptophan residues.

### Statistical analysis

The sample size of animals was chosen according to our previous experience with neurodegenerative disease mouse models. Plots were generated with GraphPad Prism v 9.0 and the Seaborn Python Library. Statistical analyses were carried out with GraphPad Prism v 9.0. For assessment of differences between two groups, unpaired Student’s *t*‐test was applied, and equal variance was confirmed with *F*‐test. Normal distribution was tested with GraphPad Prism v 9.0 and confirmed for all relevant comparisons. For datasets with multiple groups and one or more independent variables, ANOVA test including appropriate post hoc tests was used as indicated in the figure legends, and equal variance was confirmed with Brown–Forsythe and Bartlett’s test. Differences were considered statistically significant with *P* < 0.05. Data are presented as mean ± SD.

## Author contributions

MSH, FUH, RK, and ID conceived the project. SB, EKS‐T, KV, RK, and ID designed experiments. EKS‐T and KV performed proteotoxic stress experiments in cultured neurons. EKS‐T generated and characterized Fluc‐EGFP transgenic mice, and conducted luciferase assays in R6/2 mice. SB performed experiments in acute brain slices. SB and A‐LB performed luciferase assays in aging Fluc‐EGFP mice and in rTg4510 mice, and histology in old rTg4510 mice and in R6/2 mice. SB and PL performed histology in young rTg4510 mice. ID and JL performed experiments with HD94 mice. KV and A‐LB conducted experiments with neuronal cultures co‐transfected with Fluc‐EGFP and aggregating proteins. MSH and FUH provided tools and reagents. RK and ID supervised the project. SB, EKS‐T, KV, and ID designed the figures. ID wrote the manuscript with input from the other authors.

## Conflict of interest

The authors declare that they have no conflict of interest.

## Supporting information



Appendix

Expanded View Figures PDF

Source Data for Expanded View and Appendix

Source Data for Figure 1

Source Data for Figure 2

## Data Availability

This study includes no data deposited in external repositories.
