# Supplementary material for: Fluc‐EGFP reporter mice reveal differential alterations of neuronal proteostasis in aging and disease
Source: EMBO J. 2021 Aug 19;40(19):e107260. doi: 10.15252/embj.2020107260 (PMC8488555; doi:10.15252/embj.2020107260)
Supplement: Supplementary file 1 — Appendix [file EMBJ-40-e107260-s004.pdf]

Appendix

Novel reporter mouse reveals neuronal proteostasis alterations in aging and disease

Sonja Blumenstock[1,2,*], Elena Katharina Schulz-Trieglaff[1,*], Kerstin Voelkl[1,2], Anna-Lena Bolender[1,2], Paul Lapios[1,2], Jana Lindner[1], Mark S. Hipp[3,4,5], F. Ulrich Hartl[3], Rüdiger Klein[1] and Irina Dudanova[1,2]

Table of contents

Appendix Figure S1. Control experiments for proteotoxic stress treatments.

Appendix Figure S2. Further characterization of Fluc-EGFP mice.

Appendix Figure S3. Co-expression of Fluc-EGFP variants with control HTT, and analysis of subcellular localization of mHTT and β23 variants.

Appendix References

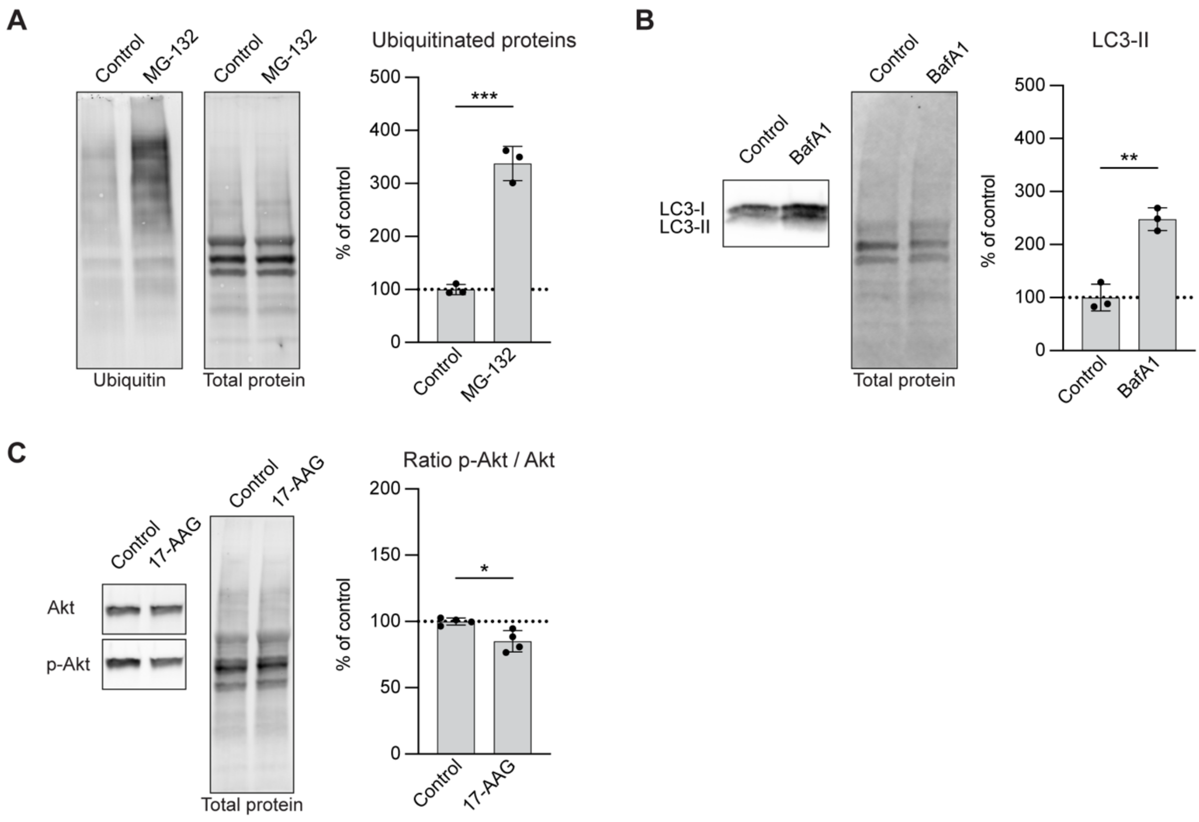

Appendix Figure S1. Control experiments for proteotoxic stress treatments.
A, Left, Western blot of primary neurons transfected with FlucWT-EGFP and treated with 5 µM MG-132 at DIV 3+2 for 4 hours. Ubiquitination levels were normalized to total protein. Right, quantification of ubiquitinated proteins in MG-132 vs. vehicle-treated lysates. N=3 biological replicates. Two-tailed t-test.
B, Left, Western blot of primary neurons transfected with FlucWT-EGFP and treated with 10 nM Bafilomycin A1 (BafA1) at DIV 3+2 for 24 hours. LC3B-II levels were normalized to total protein. Right, quantification of LC3B-II in neuronal lysates. N=3 biological replicates. Two-tailed t-test.
C, Left, Western blot of primary neurons treated with 0.5 µM 17-AAG at DIV 5 for 24 hours. Right, quantification of phospho-Akt / total Akt ratio as a readout of Hsp90 inhibition (Chen et al., 2020). N=4 biological replicates. Two-tailed t-test.
Data information: Error bars represent SD. Significance: *p<0.05; **p<0.01; ***p<0.001.

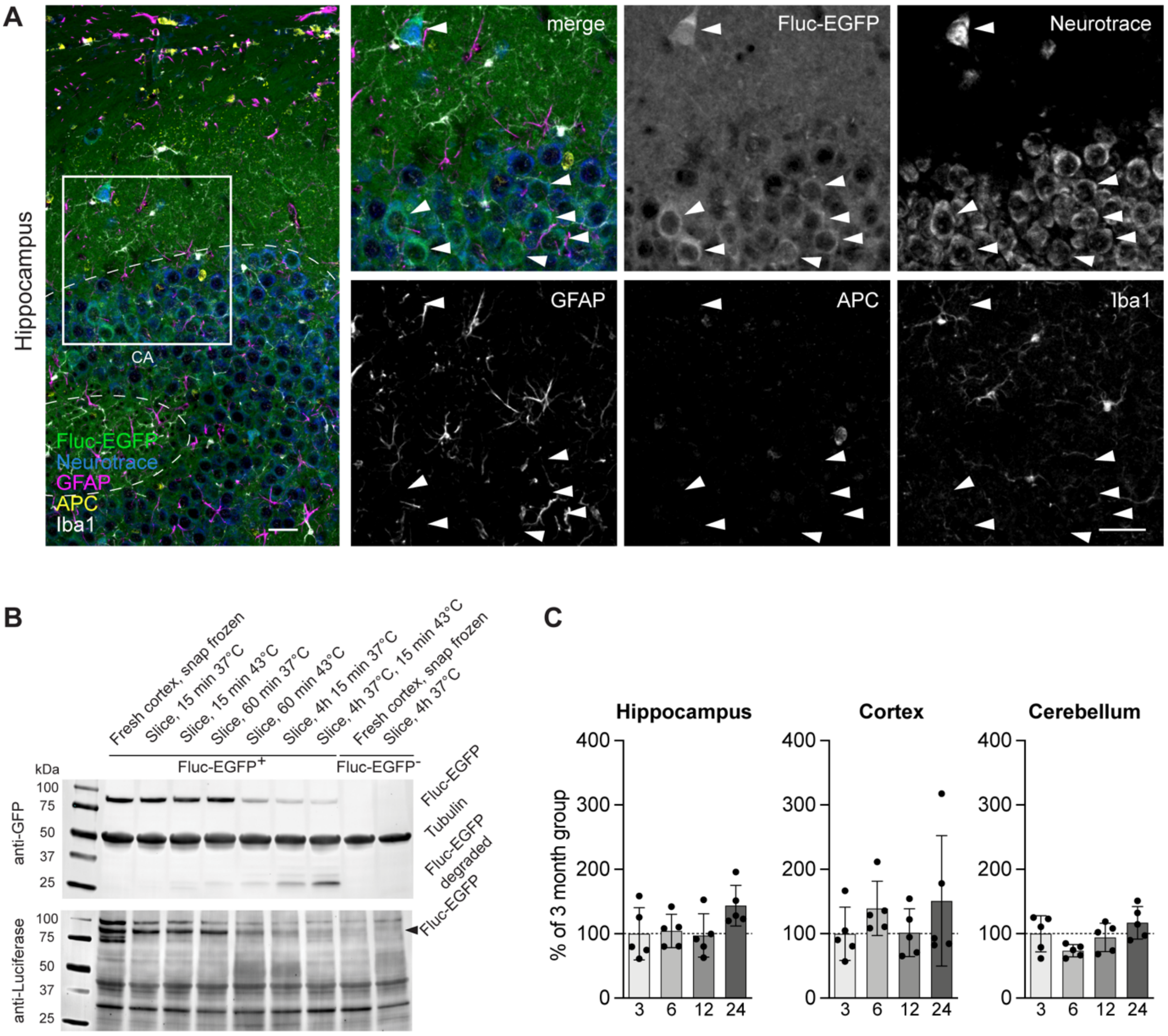

Appendix Figure S2. Further characterization of Fluc-EGFP mice.

A, Analysis of Fluc-EGFP expression in neurons and glia in the hippocampus of 3-4-month-old Fluc-EGFP mice. Brain sections were stained against GFAP (magenta), APC (yellow), and Iba1 (white). Fluc-EGFP was detected by EGFP fluorescence (green), neurons were labeled with Neurotrace (blue). Hippocampal CA layer is marked with dashed lines. Arrowheads in the higher magnification images point to Fluc-EGFP-positive cells. The experiment was repeated in N=5 animals with similar results.

B, Acute brain slices from Fluc-EGFP mice or non-transgenic controls were kept at 37°C or heat shocked at 43°C for the indicated periods of time. Western blots for GFP (top) and luciferase (bottom) are shown. Note the decrease in Fluc-EGFP band and appearance of lower molecular weight Fluc-EGFP degradation products after prolonged incubation. For all further experiments, only the GFP antibody was used, because it produced less unspecific signal than the luciferase antibody.

C, Analysis of Fluc-EGFP protein quantity in the indicated brain regions of Fluc-EGFP mice at different ages. Same data as in Fig. 2F, but normalized to total protein instead of tubulin. One-way ANOVA with Bonferroni's multiple comparisons test: not significant for all groups.

Data information: Error bars represent SD. Scale bars in A, 30 µm.

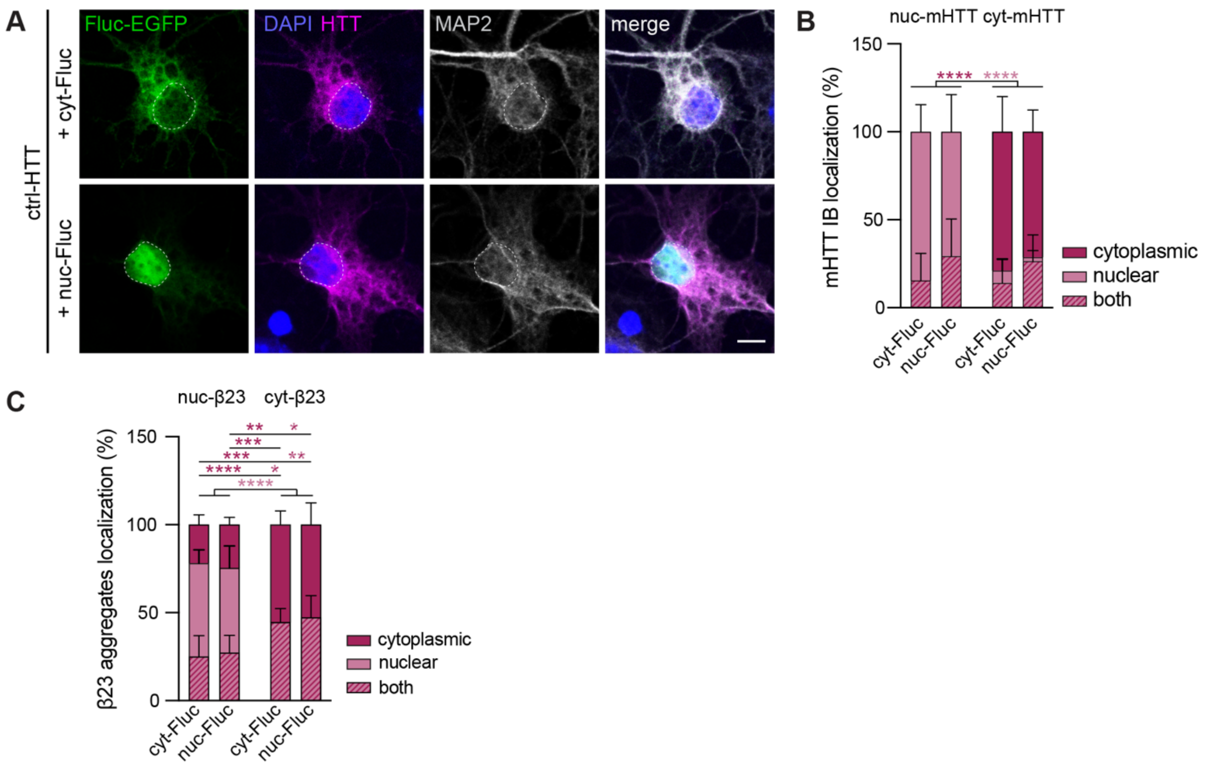

Appendix Figure S3. Co-expression of Fluc-EGFP variants with control HTT, and analysis of subcellular localization of mHTT and β23 variants.

A, Primary cortical neurons transfected with cyt-Fluc-EGFP (upper row) or nuc-Fluc-EGFP (lower row) (green), in combination with control HTT-exon1 (HTT-Q25-mCherry, magenta). Cells were fixed at DIV 3+2 and stained for the neuronal marker MAP2 (grey). Nuclei were labeled with DAPI (blue). Dashed lines mark the nuclei. Corresponding cultures transfected with mHTT constructs are shown in Fig. 5A-B.

B, Quantification of mHTT IB localization in the cytoplasm, nucleus, or both in the indicated conditions. Three-way ANOVA with Tukey's multiple comparisons test. ANOVA: Localization, **p=0.0049; HTT, n.s.; Fluc, n.s.; Localization x HTT, ****p<0.0001; Localization x Fluc, n.s; HTT x Fluc, n.s.; Localization x HTT x Fluc, n.s. Dark-red and light-red asterisks indicate significant pairwise comparisons of the fractions of cells with mHTT IBs localized in the cytoplasm and in the nucleus, respectively.

C, Quantification of β23 aggregate localization in the cytoplasm, nucleus, or both in the indicated conditions. Three-way ANOVA with Tukey's multiple comparisons test. ANOVA: Localization, **p=0.0012; β23, n.s.; Fluc, n.s.; Localization x β23, ****p<0.0001; Localization x Fluc, n.s.; β23 x Fluc, n.s.; Localization x β23 x Fluc, n.s. Dark-red, light-red and striped asterisks indicate significant pairwise comparisons of the fractions of cells with aggregates in the cytoplasm, in the nucleus, and in both compartments, respectively.

Data information: Error bars represent SD. Significance: *p<0.05; **p<0.01; ***p<0.001; ****p<0.0001. Scale bar in A, 5 µm.

Appendix References

Chen, F., Xie, H., Bao, H., Violetta, L., and Zheng, S. (2020). Combination of HSP90 and autophagy inhibitors promotes hepatocellular carcinoma apoptosis following incomplete thermal ablation. Mol Med Rep *22*, 337-343.
