# Supplementary material for: Fluc‐EGFP reporter mice reveal differential alterations of neuronal proteostasis in aging and disease
Source: EMBO J. 2021 Aug 19;40(19):e107260. doi: 10.15252/embj.2020107260 (PMC8488555; doi:10.15252/embj.2020107260)
Supplement: Supplementary file 2 — Expanded View Figures PDF [file EMBJ-40-e107260-s001.pdf]

Expanded View Figures

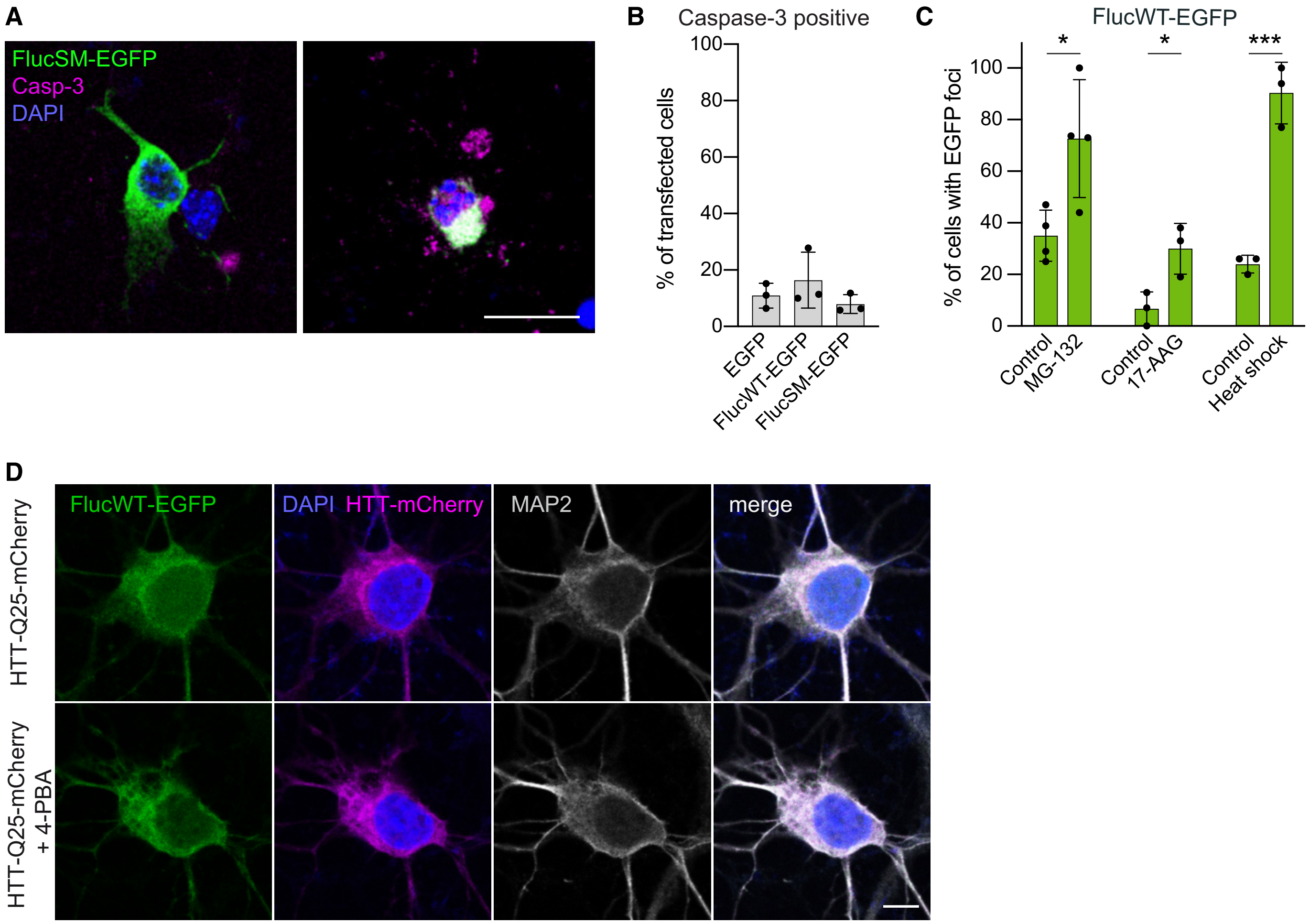

Figure EV1. Additional experiments with Fluc-EGFP in primary neurons.

- A Examples of neurons expressing FlucSM-EGFP (green) that are negative (left) or positive (right) for cleaved caspase-3 (magenta). Nuclei are labeled with DAPI (blue).
- B Quantification of the fraction of transfected neurons positive for cleaved caspase-3 at DIV 3 + 2. $N = 3$ independent experiments; one-way ANOVA. No significant differences were observed.
- C Quantification of FlucWT-EGFP foci formation in transfected neurons upon indicated treatments. $N = 3$–4 independent experiments; two-tailed t-test.
- D DIV 3 + 2 cortical neurons co-transfected with FlucWT-EGFP (green) and HTT-Q25-mCherry (magenta) and treated with 4-PBA (lower row) or vehicle control (upper row) from DIV 3. Cultured neurons were stained for MAP2 (gray) as a neuronal marker, and nuclei were labeled with DAPI (blue).

Data information: Error bars represent SD. Significance: $^{*}P < 0.05$; $^{***}P < 0.001$. Scale bars: A, 20 µm; D, 5 µm.

 © 2021 The Authors

Figure EV2. Characterization of Fluc-EGFP reporter mice.

A Analysis of Fluc-EGFP expression in neurons and glia. Cortical sections from 3 to 4-month-old Fluc-EGFP mice were stained against GFAP (magenta), APC (yellow), and Iba1 (white). Fluc-EGFP was detected by EGFP fluorescence (green), and neurons were labeled with Neurotrace (blue). Arrowheads point to Fluc-EGFP-positive cells. Dashed lines indicate borders of cortical layers. The experiment was repeated in $N = 5$ animals with similar results. Similar analysis in the hippocampus is shown in Appendix Fig S2A.

B–D Linearity of luciferase activity in tissue samples. Cortical lysates from 5 Fluc-EGFP mice (shown in shades of gray) were used in different dilutions (25, 50, 75, 100, 125 and 150 μg input protein quantity). All values were normalized to the 75 μg dilution. (B) Measured luciferase activity of the samples. (C) Measured protein quantity. (D) Linear regression of input protein quantity to specific luciferase activity (measured luciferase activity normalized to measured protein quantity) for individual mice (gray lines) and mean regression of five mice (black line); red line shows the corrected relation where 100% input protein quantity corresponds to 100% specific activity.

E Quantification of luciferase activity in Fluc-EGFP mice and non-transgenic littermates. $N = 3$ mice per genotype.

F Western blots of acute brain slices of indicated genotype treated with 5 μM MG-132 or vehicle (control) for 4 h. The part of the lane used for quantification of ubiquitin is indicated with a bracket (left). Total protein load (right, stain-free blot) was used for normalization.

G Quantification of increase in ubiquitinated proteins upon MG-132 treatment in brain slices from Fluc-EGFP mice and control littermates. $N = 5$ mice of each genotype. Green and gray asterisks indicate comparisons to the corresponding vehicle-treated control slices by one-sample *t*-test. Comparison between MG-132 treated slices of different genotypes was performed by two-tailed *t*-test.

Data information: Error bars represent SD. Significance: $^*P < 0.05$; $^{**}P < 0.01$; n.s.—not significant. Scale bars in A, 30 μm.
Source data are available online for this figure.

© 2021 The Authors

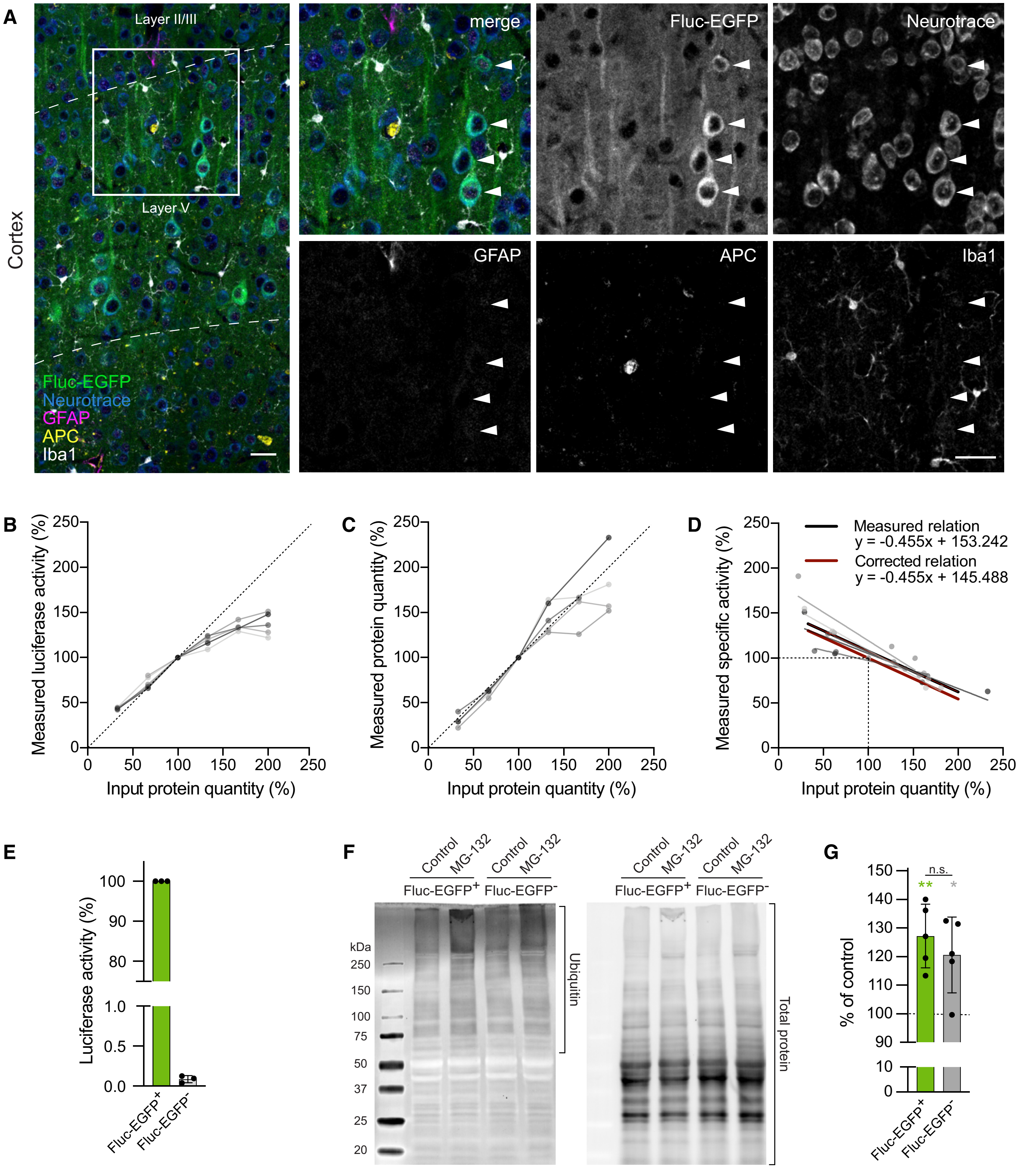

Figure EV2.

 © 2021 The Authors

Figure EV3. Additional analyses of Fluc-EGFP reporter in rTg4510 mice.

A Images of the cortex and hippocampus of control littermates (left) and rTg4510:Fluc-EGFP mice (right) stained for p-tau (AT8, magenta) at the indicated ages. Fluc-EGFP was detected by EGFP fluorescence (green), neurons were labeled with Neurotrace (blue). Higher-magnification images show the areas indicated by the boxes, with neuronal cell bodies outlined. Hippocampal CA layer is marked by dashed lines. Arrowheads point to Fluc-EGFP foci. Note that there is little colocalization between EGFP foci and p-tau.

B Representative Western blots of cortical (top) and hippocampal (bottom) lysates from 4-month-old (left) and 16-month-old (right) rTg4510:Fluc-EGFP mice and control littermates. Short bracket with an arrow indicates the high molecular weight species observed in older mice. Long bracket indicates the part of the lane that was used for Fluc protein quantification.

Data information: Scale bars in A, 10 µm.
Source data are available online for this figure.

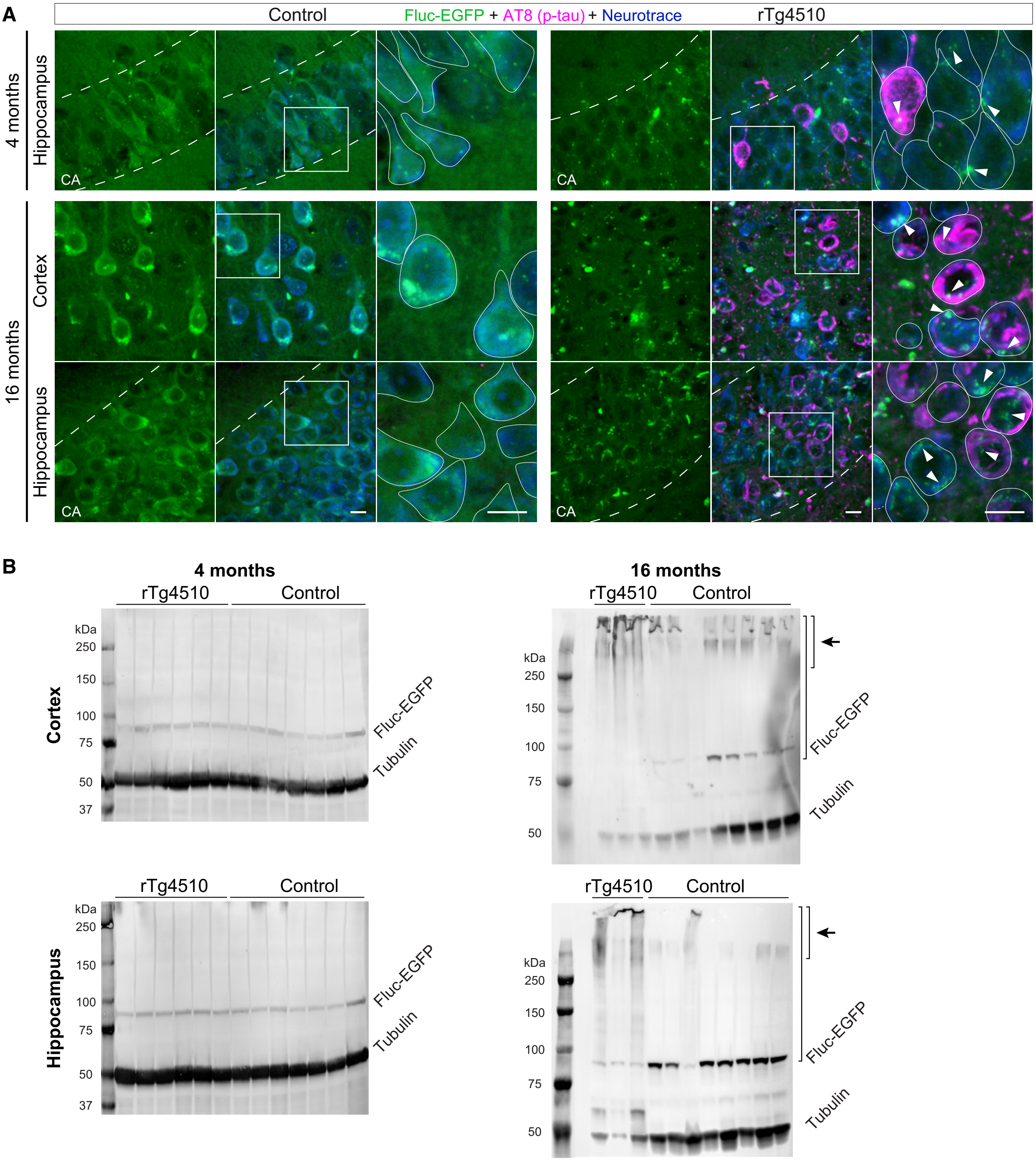

Figure EV3.

© 2021 The Authors

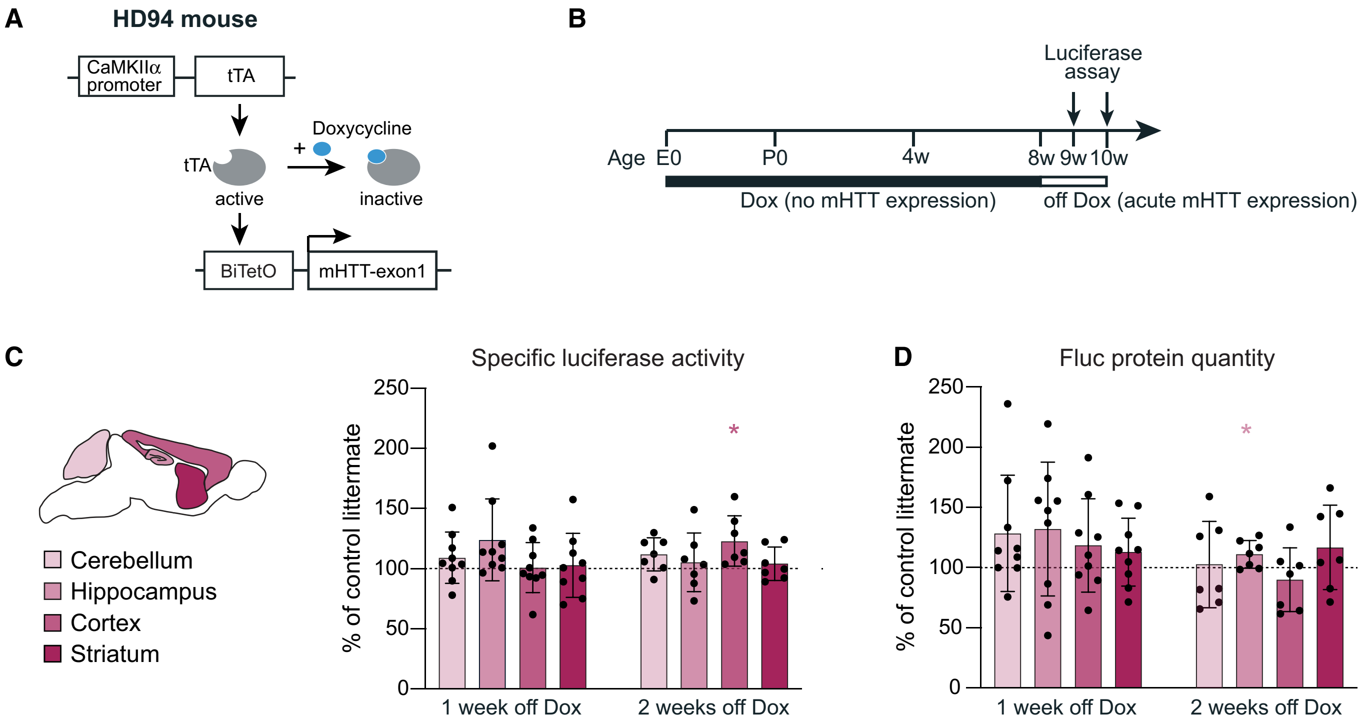

Figure EV4. Proteostasis measurements in HD94:Fluc-EGFP mice upon acute induction of mHTT expression.

A Scheme of the HD94 transgenic strategy.

B Experimental timeline. E0, embryonic day 0; P0, postnatal day 0.

C, D Luciferase assay in HD94 mice. (C) Left, scheme of brain regions used for luciferase assay color-coded by their vulnerability to Huntington's disease. Right, specific luciferase activity of cerebellar, hippocampal, cortical, and striatal tissue lysates. (D) Fluc-EGFP protein levels measured by Western blot. WT:Fluc-EGFP, CaMKIIα-tTA:Fluc-EGFP, and BiTetO:Fluc-EGFP littermates did not differ from each other and were pooled together as controls. Values of HD94:Fluc-EGFP mice were normalized to their respective pooled littermate controls. 1 week off Dox: $N = 9$ HD94:Fluc-EGFP mice; 5 CaMKIIα-tTA:Fluc-EGFP mice; 7 BiTetO:Fluc-EGFP mice; and 7 WT:Fluc-EGFP mice; 2 weeks off Dox: 7 HD94:Fluc-EGFP mice; 2 CaMKIIα-tTA:Fluc-EGFP mice; 3 BiTetO:Fluc-EGFP mice; and 3 WT:Fluc-EGFP mice. Colored asterisks in C and D indicate significant comparisons to the respective brain regions of control littermates (one-sample *t*-test).

Data information: Error bars represent SD. Significance: $*P < 0.05$.

Figure EV5. Response of nuclear and cytoplasmic Fluc-EGFP to nuclear and cytoplasmic β23 protein.

A, B Primary cortical neurons transfected with cyt-Fluc-EGFP (A) or nuc-Fluc-EGFP (B) (green), in combination with nuc-β23 (upper rows) or cyt-β23 (lower rows). Cells were fixed at DIV 3 + 2 and stained for myc to detect β23 (magenta) and for the neuronal marker MAP2 (gray). Nuclei were labeled with DAPI (blue). Arrowheads point to Fluc-EGFP foci. Dashed lines mark the nuclei. Schemes on the right summarize the cellular distribution of the respective versions of Fluc-EGFP (green) and β23 (magenta).

C Quantification of Fluc-EGFP foci formation. $N = 3$–4 independent experiments. Two-way ANOVA with Tukey's multiple comparisons test. ANOVA: β23, n.s.; Fluc, $****P < 0.0001$; β23 × Fluc, n.s. Significant pairwise comparisons are indicated on the graph.

D Quantification of the subcellular localization of the respective Fluc-EGFP protein in the presence of the indicated β23 versions. $N = 3$–4 independent experiments. Three-way ANOVA with Tukey's multiple comparisons test. ANOVA: Localization, $****P < 0.0001$; β23, n.s., Fluc, n.s.; Localization × β23, n.s.; Localization × Fluc, $****P < 0.0001$; β23 × Fluc, n.s.; Localization × β23 × Fluc, $*P = 0.0135$. Dark-green and light-green asterisks indicate significant pairwise comparisons of the fractions of cells with Fluc-EGFP localized in the cytoplasm or in the nucleus, respectively.

E Quantification of cyt-Fluc-EGFP foci localization in the presence of nuc-β23 or cyt-β23. $N = 3$–4 independent experiments. Two-way ANOVA with Bonferroni's multiple comparisons test. ANOVA: Localization, $****P < 0.0001$; β23, n.s.; Localization × β23, n.s.

Data information: Error bars represent SD. Significance: $****P < 0.0001$. Scale bar in A, B, 5 μm.

© 2021 The Authors

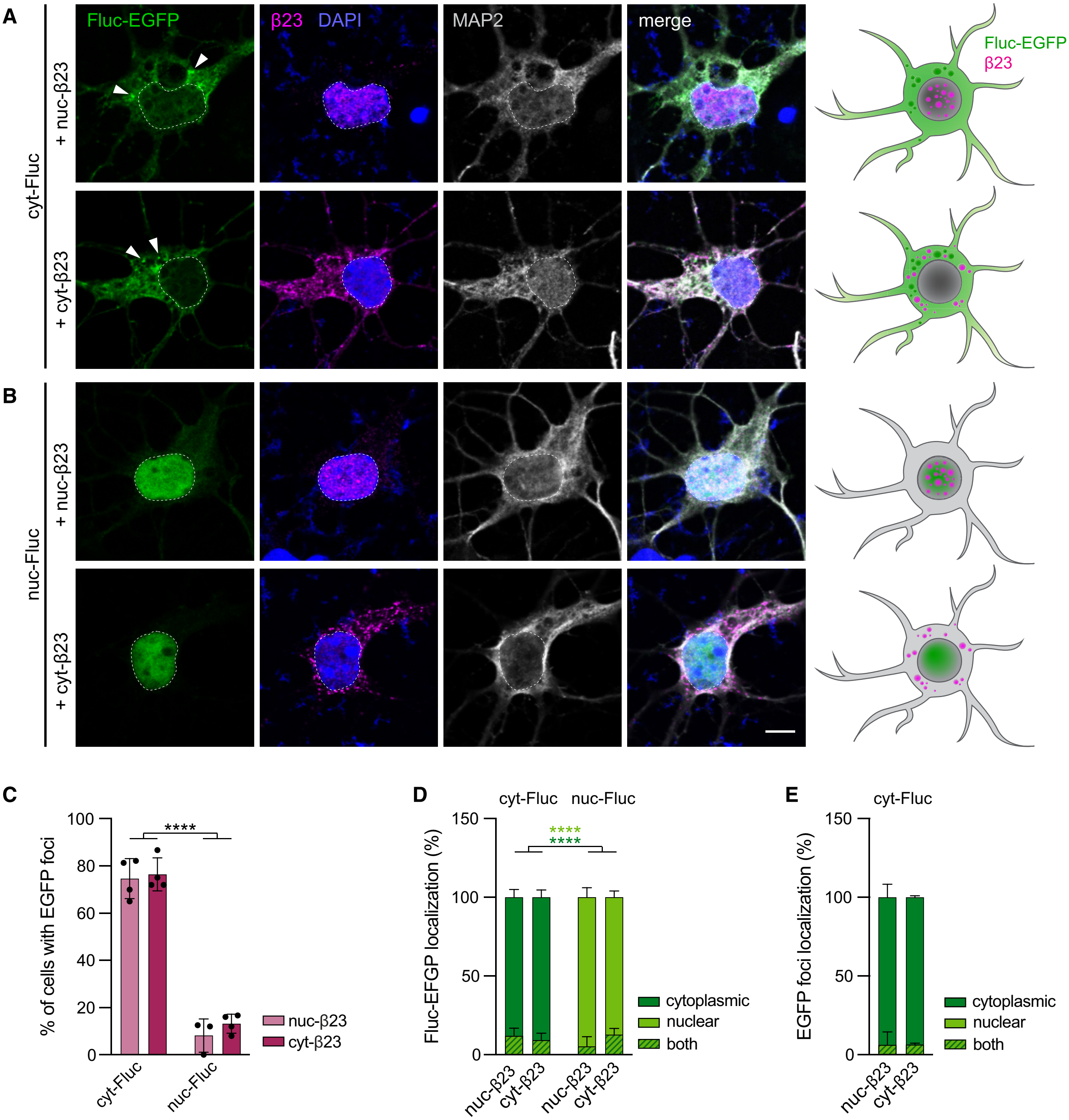

Figure EV5.

 © 2021 The Authors
