# Supplementary figures and images for: Fluc‐EGFP reporter mice reveal differential alterations of neuronal proteostasis in aging and disease
Source: EMBO J. 2021 Aug 19;40(19):e107260. doi: 10.15252/embj.2020107260 (PMC8488555; doi:10.15252/embj.2020107260)

Source Data for Fig EV2

F

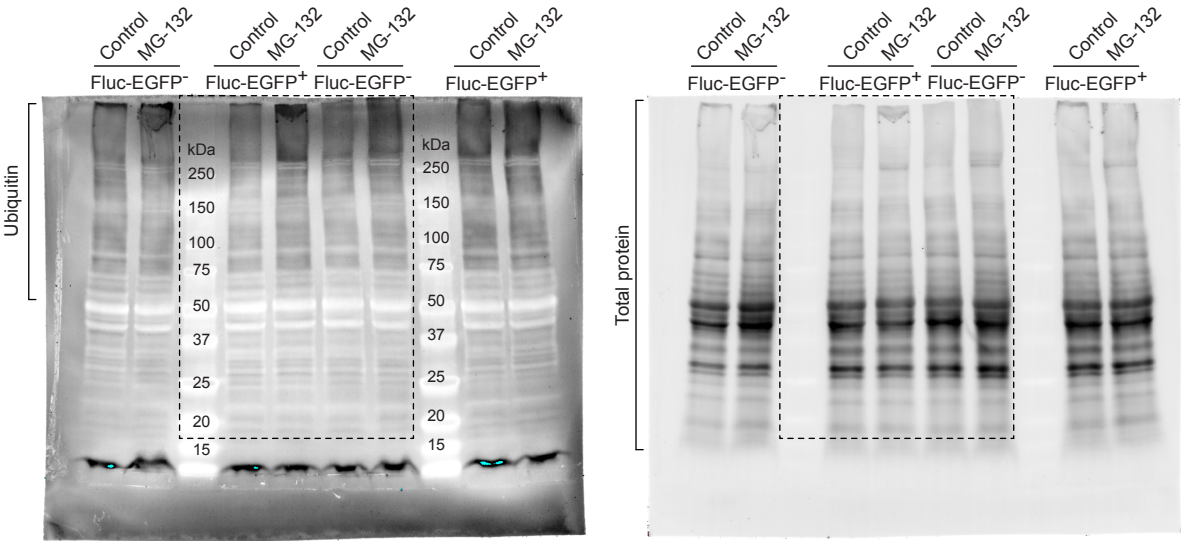

Supplement: Supplementary file 3 — Source Data for Expanded View and Appendix [file EMBJ-40-e107260-s006.zip › source_data_EV_Supp/source_data_EV2.pdf]

### Source Data for Fig EV3

# B

**4 months**

**16 months**

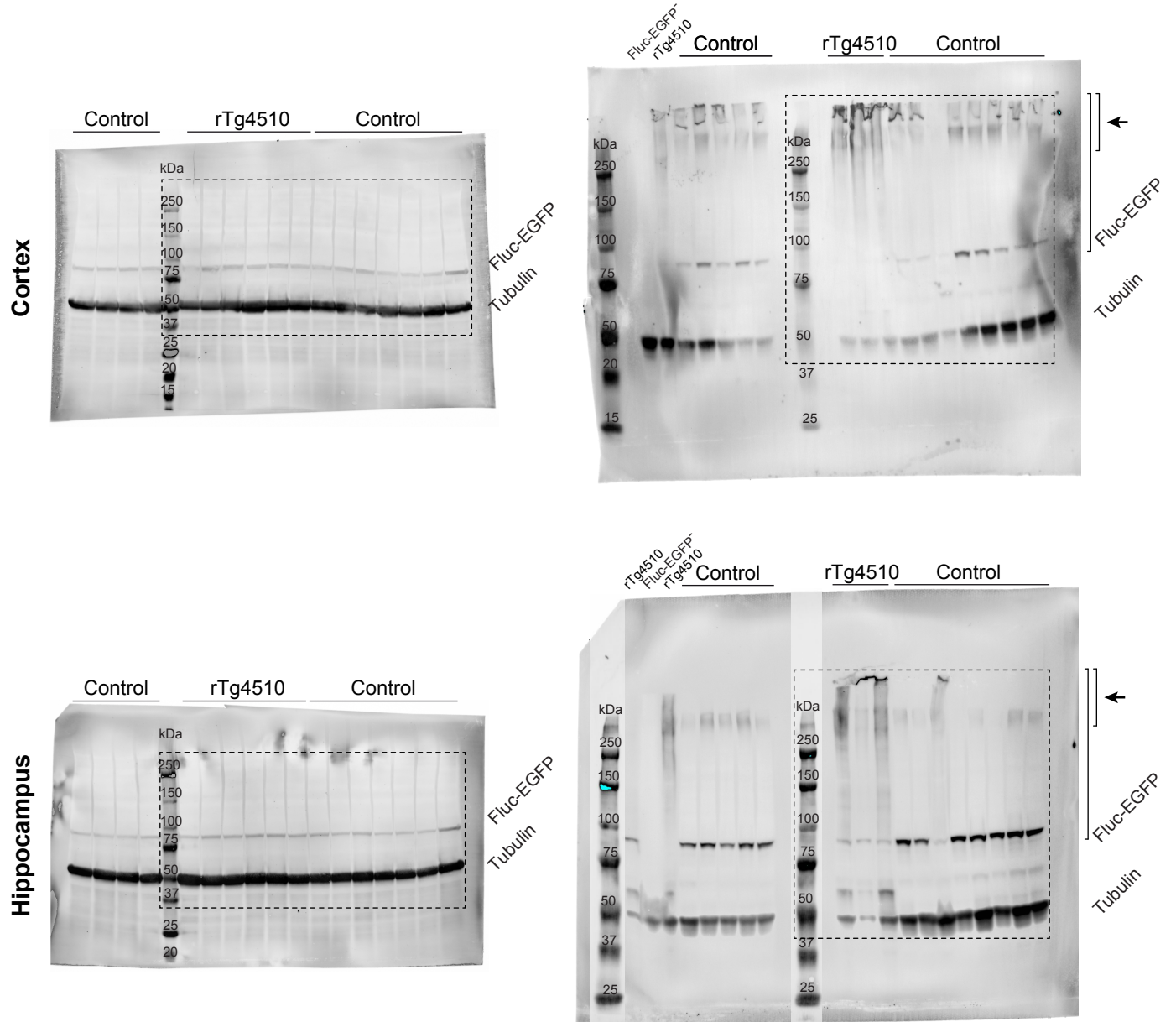

Supplement: Supplementary file 3 — Source Data for Expanded View and Appendix [file EMBJ-40-e107260-s006.zip › source_data_EV_Supp/source_data_EV3.pdf]

## Source Data for Fig S1

**A**

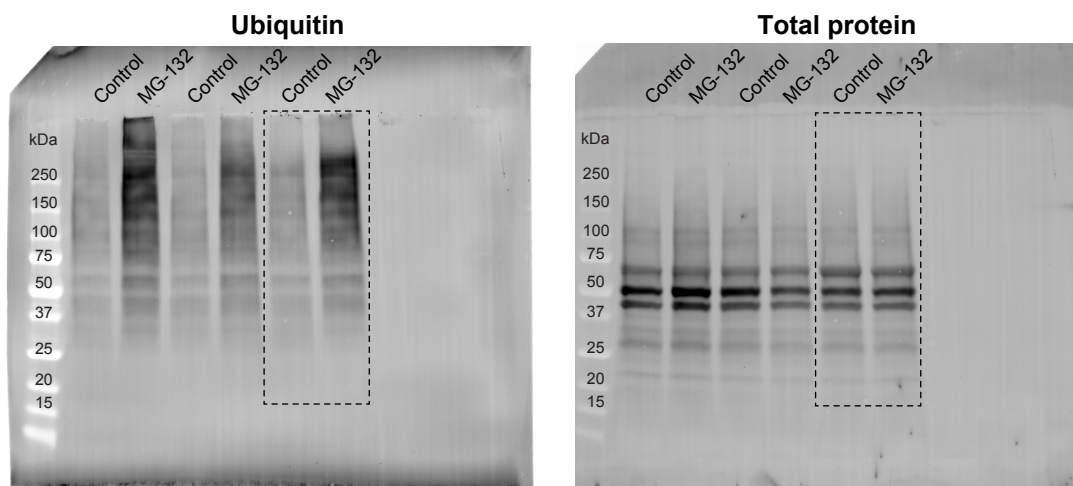

**B**

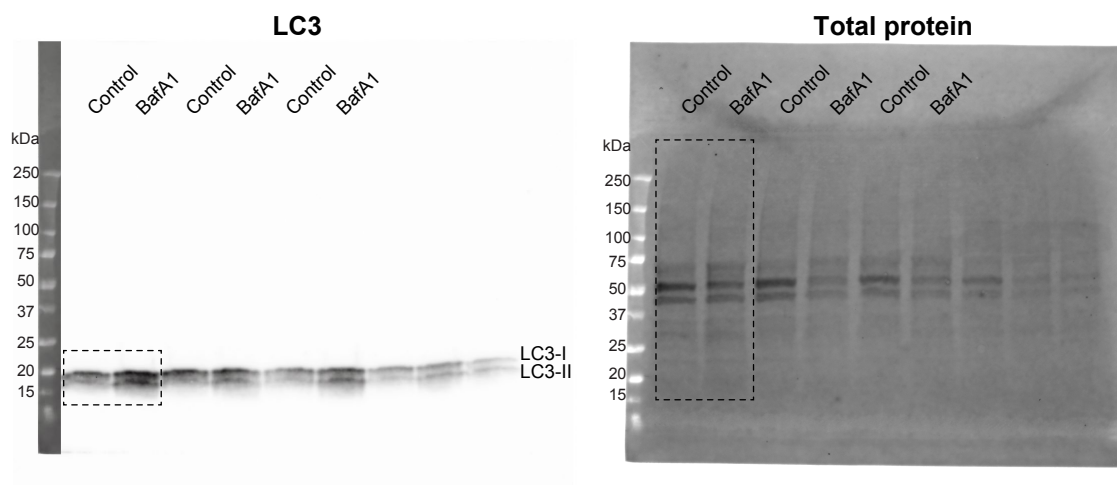

**C**

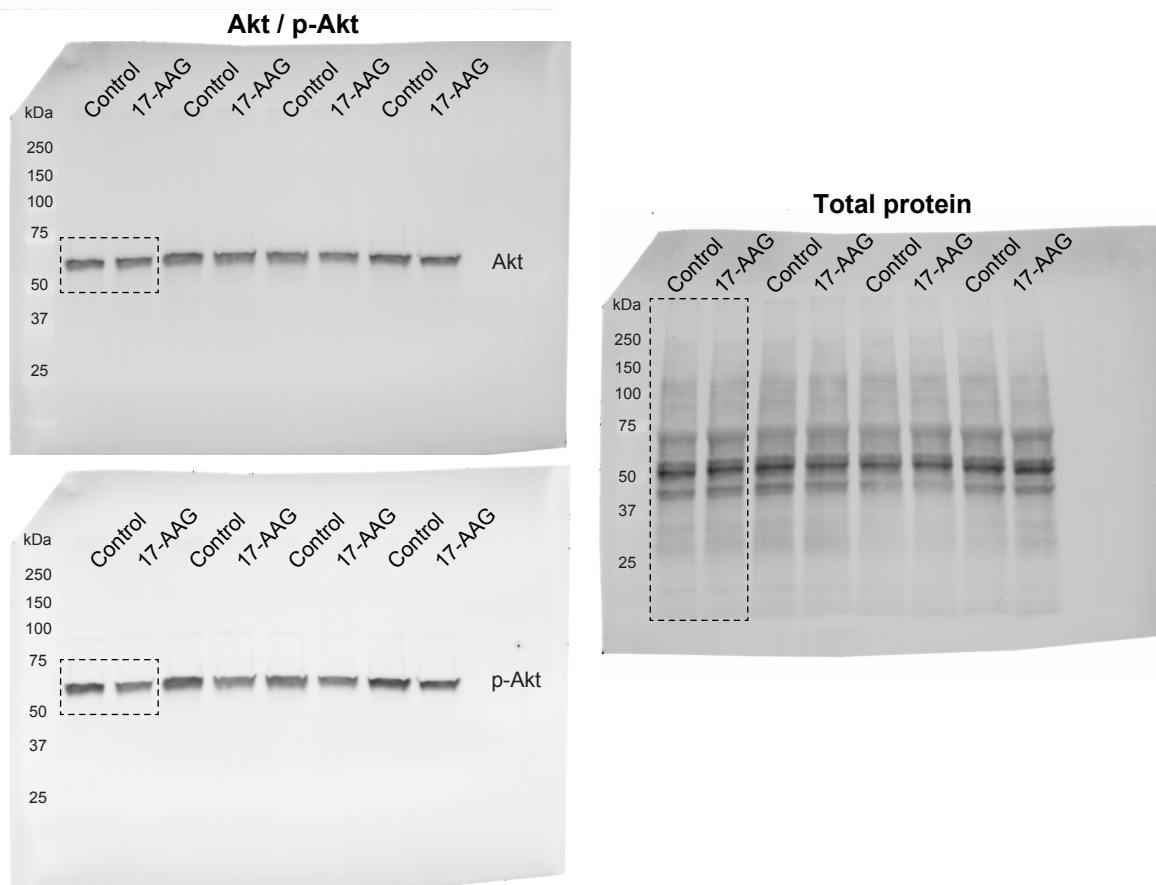

Supplement: Supplementary file 3 — Source Data for Expanded View and Appendix [file EMBJ-40-e107260-s006.zip › source_data_EV_Supp/source_data_S1.pdf]

## Source Data for Fig S2

**B**

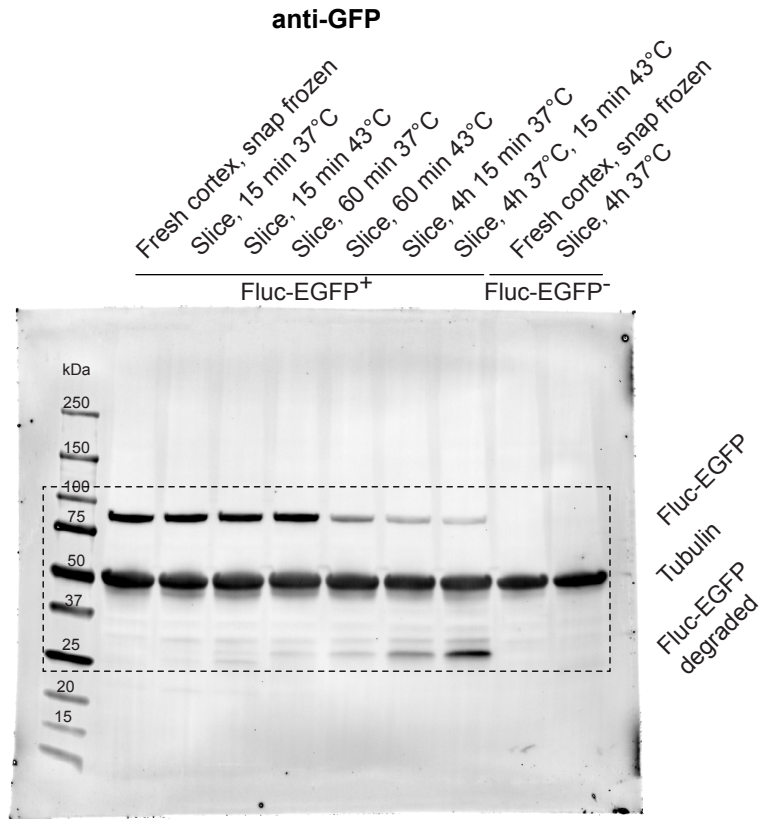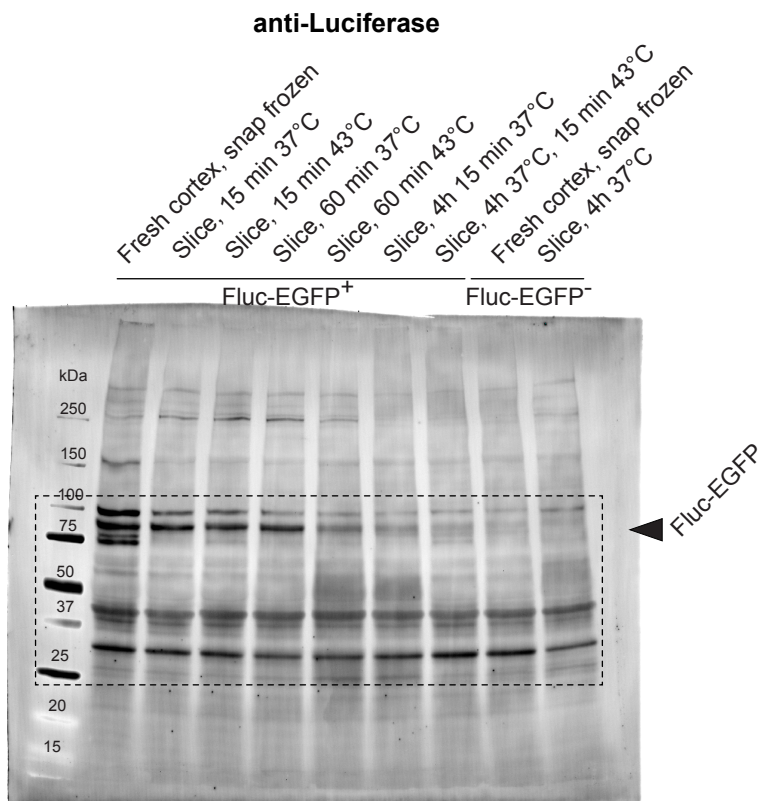

Supplement: Supplementary file 3 — Source Data for Expanded View and Appendix [file EMBJ-40-e107260-s006.zip › source_data_EV_Supp/source_data_S2.pdf]

## Source Data for Fig 1

**C**

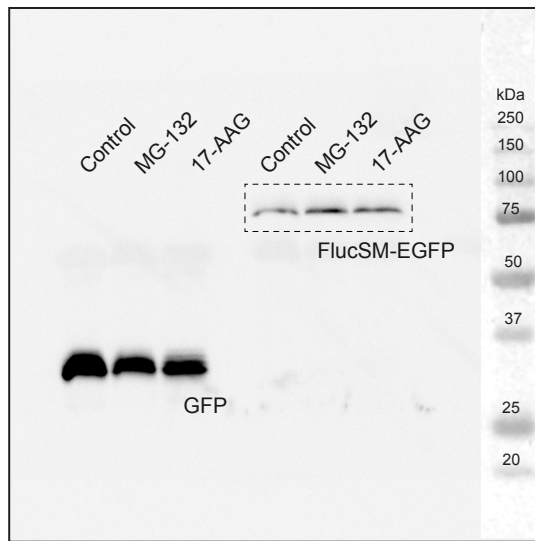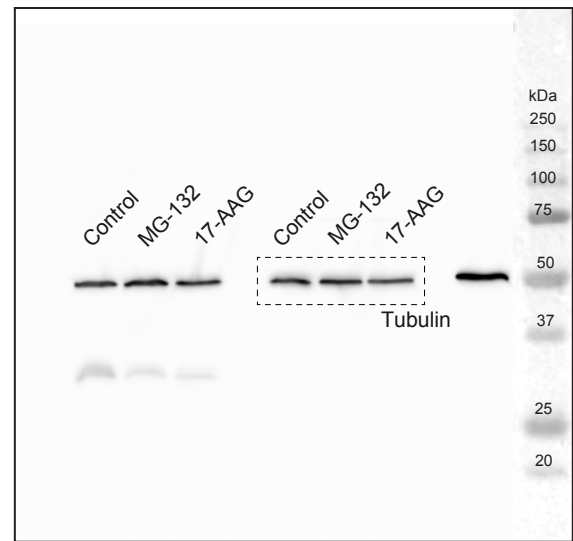

Supplement: Supplementary file 4 — Source Data for Figure 1 [file EMBJ-40-e107260-s002.pdf]

## Source Data for Fig 2

**C**

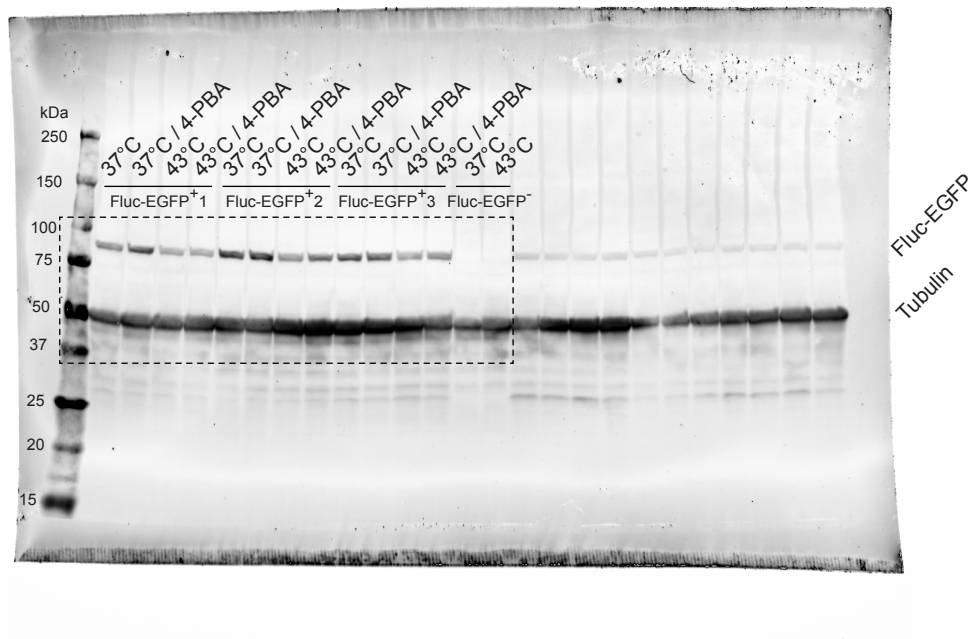

**D**

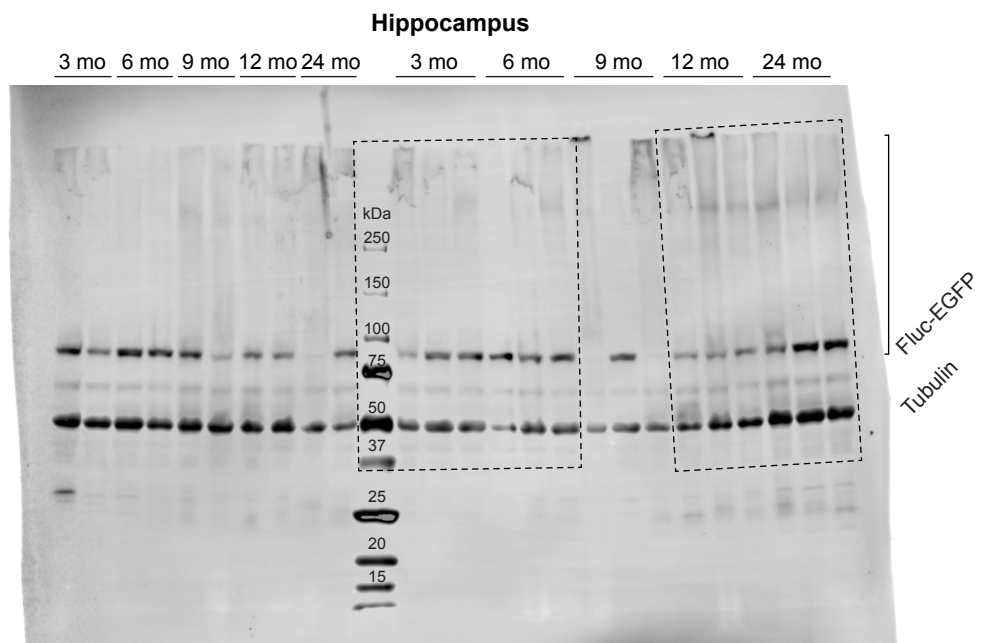

Supplement: Supplementary file 5 — Source Data for Figure 2 [file EMBJ-40-e107260-s005.pdf]
