# Supplementary material for: Fluc‐EGFP reporter mice reveal differential alterations of neuronal proteostasis in aging and disease
Source: EMBO J. 2021 Aug 19;40(19):e107260. doi: 10.15252/embj.2020107260 (PMC8488555; doi:10.15252/embj.2020107260)
Supplement: Supplementary file 6 [file EMBJ-40-e107260-s003.pdf]

THE EMBO JOURNAL

Novel reporter mouse reveals neuronal proteostasis alterations in aging and disease

Sonja Blumenstock, Elena Katharina Schulz-Trieglaff, Kerstin Voelkl, Anna-Lena Bolender, Paul Lapios, Jana Lindner, Mark Hipp, F. Ulrich Hartl, Rudiger Klein, and Irina Dudanova
DOI: 10.15252/embj.2020107260

Corresponding author: Irina Dudanova (idudanova@neuro.mpg.de)

Review Timeline:

Submission Date:	9th Nov 20
Editorial Decision:	18th Dec 20
Revision Received:	31st May 21
Editorial Decision:	28th Jun 21
Revision Received:	9th Jul 21
Accepted:	19th Jul 21

Editor: Karin Dumstrei

Transaction Report:

(Note: With the exception of the correction of typographical or spelling errors that could be a source of ambiguity, letters and reports are not edited. Depending on transfer agreements, referee reports obtained elsewhere may or may not be included in this compilation. Referee reports are anonymous unless the Referee chooses to sign their reports.)

1st Editorial Decision 18th Dec 2020

Hi Irina,

Thank you for submitting your manuscript to The EMBO Journal. Your manuscript has now been seen by three referees and their comments are provided below.

As you can see below, the referees find the analysis interesting and appreciate the resource value of the developed reporter mice. However, they also raise important concerns that needs to be resolved for further consideration here. Should you be able to extend the analysis along the lines indicated by the referees then I would like to consider a revised version.

I am happy to discuss the raised points further and maybe it would be most helpful to do so via phone or video. I will contact you in the next few days to discuss this further.

When preparing your letter of response to the referees' comments, please bear in mind that this will form part of the Review Process File, and will therefore be available online to the community. For more details on our Transparent Editorial Process, please visit our website: https://www.embopress.org/page/journal/14602075/authorguide#transparentprocess

We generally allow three months as standard revision time. As a matter of policy, competing manuscripts published during this period will not negatively impact on our assessment of the conceptual advance presented by your study. However, we request that you contact the editor as soon as possible upon publication of any related work, to discuss how to proceed. Should you foresee a problem in meeting this three-month deadline, please let us know in advance and I can grant an extension.

Thank you for the opportunity to consider your work for publication. I look forward to your revision.

with best wishes

Karin

Karin Dumstrei, PhD
Senior Editor
The EMBO Journal

Instructions for preparing your revised manuscript:

Please make sure you upload a letter of response to the referees' comments together with the revised manuscript.

Please also check that the title and abstract of the manuscript are brief, yet explicit, even to non-specialists.

When assembling figures, please refer to our figure preparation guideline in order to ensure proper formatting and readability in print as well as on screen:
https://bit.ly/EMBOPressFigurePreparationGuideline

IMPORTANT: When you send the revision we will require
- a point-by-point response to the referees' comments, with a detailed description of the changes made (as a word file).
- a word file of the manuscript text.
- individual production quality figure files (one file per figure)
- a complete author checklist, which you can download from our author guidelines (https://www.embopress.org/page/journal/14602075/authorguide).
- Expanded View files (replacing Supplementary Information)
Please see out instructions to authors
https://www.embopress.org/page/journal/14602075/authorguide#expandedview

Please remember: Digital image enhancement is acceptable practice, as long as it accurately represents the original data and conforms to community standards. If a figure has been subjected to significant electronic manipulation, this must be noted in the figure legend or in the 'Materials and Methods' section. The editors reserve the right to request original versions of figures and the original images that were used to assemble the figure.

Further information is available in our Guide For Authors:
https://www.embopress.org/page/journal/14602075/authorguide

The revision must be submitted online within 90 days; please click on the link below to submit the revision online before 18th Mar 2021.

Link Not Available

Referee #1:

EMBOJ-2020-107260
In this manuscript, Blumenstock et al extended previous studies from Firefly luciferase protein and its capacity as a sensor to monitor proteostasis capacity, now in this case in the central nervous system. Here, a new reporter mice carrying EGFP-fused firefly luciferase (Fluc-EGFP) is developed. The overexpressed product is an unstable protein that demands chaperone function for proper folding and reacts to proteotoxic stress by forming intracellular foci and reduced luciferase activity. Authors provide evidence of neuronal expression for this construct in response to the Prp promoter and evaluate the activation of the FLuc-EGFP system evaluating their results as proteostasis impairment in both tauopathy and Huntington's mice models. Overall, this manuscript is logic and straightforward, while it approaches properly to the mouse model and the function of the proposed system. However, a major concern along the data presented for this new transgenic mice model is the absence of key controls that demonstrate the establishment of the proteostasis impairment across figures. However, we believe that authors can easily solve this aspect. In addition, minor aspects in data or the interpretations are discussed. The idea of Fluc-GFP as a proteostasis monitor tool is a general concept and not a novelty in the field; however, this work proposes a novel animal model to monitor proteostasis capacity in the central nervous system. I list below major and minor points to improve the study, our requirements for publishing in EMBO Journal.

1. Western blot against proteotoxic stress controls are required as supplementary information in order to demonstrate the appropriate induction in response to every treatment for Fig. 1A (positive controls after MG-132, bafilomycin, 17-AAG and heat shock). Protein synthesis, autophagy markers, Hsp90 activity and HSP activation should be shown in some way respectively.
2. It has been reported that prion promoter activity decreases with aging in frontal cortex and hippocampal neurons (and indeed this doesn't happen in familial Alzheimer's disease, Whitehouse et al., 2010 J Alzheimers Dis). Protein abundance experiments are not as clear/clean as expected, regarding variability in the housekeeping control and low experimental number (western blots show only 3 samples per group). To solve this, please confirm the abundance of the housekeeping control with another control protein (GAPDH?) by western blot and also, aging experiment must be compared to Fluc-EGFP mRNA levels (a correlation study between Fluc-EGFP mRNA levels and luciferase activity also is an option). Furthermore, the comparison of wildtype littermates in the western blot is needed as basic evidence of this new transgenic mouse model.
3. In addition, experiments of luciferase activity in Figure 2 lack of control from wildtype mice, since this is the first approach of this model.
4. I think that the sentence "...suggesting that only cytoplasmic protein aggregates cause a disturbance of neuronal proteostasis" is not close enough to be proposed as a suggestion with the available data showed here. The suggestion maybe also: this protein is unable to form nuclear inclusions due to a technical issue (for example, a difficulty to translocate). For example, under the cellular Htt-97Q model (nuclear IB), the FlucSM-EGFP sensor is able to detect nuclear inclusion bodies, whereas Fluc-GFP is not (Gupta et al., 2011 Nat Methods). In addition, this mutant Huntingtin model shows signatures of ER stress, both in cell lines and in mouse model (Hyrskyluoto et al., 2014 Hum Mol Gen) confirming proteostasis alterations. The sentence proposed is overstated and I suggest adjusting it to the presented evidence. In addition, I suggest that intracellular compartments studies must be performed. For example, the re-distribution of NLS-Fluc into the cytosol reveals that there may be some modifications of the protein that does not allow its translocation. In addition, the subtitle "Cytoplasmic, but not nuclear, protein aggregates cause proteostasis impairments" is overstated and could be replaced for "Cytoplasmic, but not nuclear, protein aggregates are detected by Fluc-EGFP reporter".
5. The model proposes changes in the folding stability of Fluc-EGFP due to proteostasis impairment across the figures. However, as the results are presented, the absence of Fluc-EGFP inclusions does not mean so. Several other possibilities may occur such as the decrease of fluorescent signal or even conformational changes of Fluc protein and furthermore changes in luciferase activity may occur. A major concern throughout the data presented here is a general lack of proteostasis stress readouts elucidating such alterations. If this proposal is true, a proteostasis reestablishment experiment should solve this question. For example, a rescue experiment with chaperones overexpression (or by using chemical chaperones) should reestablish the correct folding/activity of Fluc-EGFP in proteostasis alterations context. Without this experiment, this article may be published in a methodologic journal. In my opinion, and for the interest of the Editor, at least one in vitro and one in vivo proposal of proteostasis rescue must be demonstrated here for publishing in EMBO Journal.

Referee #2:

General

The manuscript by Blumenstock et al describes the in vivo use of a well-known proteostasis reporter construct. A transgenic mouse line is constructed and crossed with 2 models of

neurodegeneration, a tau line and a Huntington line, and the behaviour of the reporter in both is compared. Given the marked difference in response of the reporter in both models, the authors move to a cell-culture based comparison of cytoplasmic and nuclear aggregates, from which they conclude that nuclear aggregates are in general less burdensome for the proteostasis network.

The work is very interesting and the use of the reporter in vivo in models of aggregation disease is highly relevant, so I am supportive of publication. I have however a number of concerns that I think should be cleared up.

Main concerns

1. The proteostasis network is notoriously highly adaptive and thus the use of a transgenic line where the sensor is present during development is potentially fraught with complications since a new homeostatic point may be reached through an adaptive response that incorporates the sensor (itself a burden). I would be much less worried if an inducible construct had been used, switched on just before readout. Given that the model is what it is, can the authors provide data to show that this is not a concern, or otherwise include clear disclaimers to this potential pitfall? I am worried that if widely used in the field, it may generate red herrings.

2. The authors generalise the difference in response between HTT and Tau, to a general difference between aggregates in the nuclear and cytoplasmic compartments. However, the sequence composition flavour of both proteins is also completely different and I dont think that the cell culture work done is conclusive to support these claims. Cultured cells have everything in abundance and probably dont model proteostatic aging very well at all, we don't know what would happen with other proteins in nuclear aggregates in a mouse model over time and we cannot infer this from cell culture work. This part of the conclusion should at least be toned down in the abstract I think.
Can the authors exclude that the sensor is simply more sensitive to cytoplasmic perturbations? or that there is a delay before nuclear stress is picked up by the sensor (older age or in cell culture terms, longer incubation times)? How does the sensor respond to p53 aggregation eg. or something in the ER, like CFTR?

Specific comments:
- In the tau model: the sensor is positive in the absence of tau hyperphosphorylation: the authors suggest this is because the proteostatic burden precedes the formation of insoluble aggregates and I think this is plausible, but it would be nice to have more certainty. For example: is there an increased staining for pFTAA in these cells? (or a similar sensor, they are avialable with a wide range of spectral properties)

-Figure 2 panels C-D; E-F and G-H:
I think it would be a more correct way to analyse if you would normalise the luciferase activity to the protein concentration for each individual measurement. Actually, exactly how it was done in Figure 1C. On top of that, how is the protein quantity determined with a smear like in the conditions 12 mo 24 mo (2I), as it looks like there is quite a reduction in protein quantity in 12 mo, which is not visualised in the bar plot in Fig 2F
The same comment applies for Figures 3F-G and 4B-C

- Figure 5
An obvious extra control would be to target the p-tau from rTg4510 mice to the nucleus. The

sensor should not be detecting those inclusions. If it does, it is not the compartment that matters but the identity of the aggregate.

- Final sentence of results on page 9: "In summary, the Fluc-EGFP sensor reacts only to the presence of cytoplasmic, but not nuclear, protein aggregates, suggesting that only cytoplasmic protein aggregates cause a disturbance of neuronal proteostasis."
The authors use one specific reporter here (Fluc), which, as stated, depends on chaperones for proper folding etc. The final statement made by the authors might be too general in the sense that if another sensor would be used, that depends on another set of chaperones, it might be possible to detect nuclear aggregate stress as well? Stating that only cytoplasmic protein aggregates cause a disturbance of neuronal proteostasis might be too broad?
In the same line: a sentence in the discussion: "proteostasis was largely intact in HD models". Not strictly correct: the reporter line does not detect it, which does not mean it's not there.

Minor concerns and suggestions

Given the widespread availability of single cells RNA sequencing techniques, I think the big opportunity in these models would be to analyse the cell-type specificity of the cells in which the reporter shows proteostatic decline.

What would really drive the adoption of this reporter in the field would be an AAV based construct with a brain tropism or other that can be introduced in any neurodegenerative disease model at any desired age. This would also overcome the issue of adaptive responses.

Referee #3:

EMBOJ-2020-107260 review.20201210

Proteostasis is known to be impaired in neurodegenerative diseases generating a vicious cycle by which the protein quality control system is being overloaded by protein aggregates.
Here, a luciferase read-out of cytoplasmic aggregation and reduced activity (wild-type and aggregation-prone mutant) was tested in vitro followed by generating transgenic mice driven by the PrP promoter. Decline in luciferase activity was found with ageing. By crossing the parental strain onto tau and HTT mutant backgrounds it was found that tau (being cytoplasmic) impaired the proteosome (reporter) whereas huntingtin (being nuclear) did not. Readouts were luciferase foci and reduced bioluminescence. A cross-over design in cell culture then showed that cytoplasmically localized Htt is capable of impairing the proteosome. The authors conclude (as stated in the abstract) that cytoplasmic, but not nuclear aggregates cause defects of cellular protein quality control.

The introduction is concise focusing on what is relevant for this particular study. The results section however lacks critical information such as age groups studied, brain areas and cell-types analyzed etc. I consider the data as interesting and the tools advancing the field, but the in vivo analysis needs to be worked over as outlined below. The discussion should go into the limitations around the in vivo models (see below).

Page 5/Fig 2A,B: Histological analysis of parental transgenic strain (PrP-FlucWT-EGFP): What is meant by salt-and-pepper-like distribution of expression? The transgenic product seems to be distributed into the soma and dendrites, exclude the nucleus, and expression is particularly strong in cortex and hippocampus. Is there any glial expression (as one would expect for the PrP promoter)?

Page 6/Fig 2C-H: Decline of luciferase expression with ageing: I suggest swapping panels H and I as H is the quantification of I.

The blot (2I) needs to be repeated as this is a composite blot!

The loading control needs to be revisited: the tubulin signal at 24 mo is much stronger than at 12 mo which makes me sceptical about the increase in Fluc protein with ageing. The MW needs to be indicated and the blot should also be probed for Fluc. For the 12 mo samples some protein is stuck in the well. The pockets should be shown for all lanes. Also, it should be indicated on the blot what has been used for quantification. (same for Fig S3 where there is a HMW smear also for the control)

Page 6/Fig 3: Crossing with rTg4510 tau mice: It is difficult to compare these data with the situation in the parental mouse strain in Fig 2 as a different magnification is shown, the nuclei are not shown, and it is not even clear in which brain area the images have been captured. The AT8 distribution looks unfamiliar to me. I am surprised that the CA layer of the hippocampus has not been used, with its typical cytoarchitecture. I am not disputing that more foci form in the crossed triple tg mice but the data presentation is unfortunate.

In 3E the authors claim that the % of cells with EGFP foci of AT8+ phospho-tau cells is the same as in AT8- phospho-tau cells. They conclude that 'Fluc-EGFP is sensitive to protein aggregation in the cytoplasm prior to formation of insoluble aggregates'; however, the authors don't state (as far as I can tell) how old the mice were when the images were taken and whether the AT8- phospho-tau cells express tau at all. They could use a pan-human tau antibody to show.

Page 7/Fig 4: Please specify in legend 4 that EM48 detects huntingtin. Huntingtin accumulates in the nucleus. Does Huntingtin 'see' the proteosome? I understand that there is a lot of literature claiming and showing proteasomal impairment in HD and HD models. How is this being reconciled? The mice (2 models) were only 3 months old (or younger). Is it possible that the pathology is simply not advanced enough, especially as for tau pathology a model was chosen that has been challenged because of a massive overexpression and an (FGF14) integration artefact. To me it is difficult to compare the tau and Htt models andthe discussion should critically discuss the models and to which extent the data can be generalized. I understand that a lot work has gone into establishing these mice and am not expecting a second tau model being tested but the discussion should discuss the limitations.

Page 8/Fig 5 is the logical experiment that comes to mind after having seen the in vivo data. By targeting HTT to the cytoplasm proteostasis impairments are being induced. All controls were done of nuclear and cytoplasmic targeting of HTT and the reporter. As a minor point for someone not working in the Huntington's space it might be worthwhile mentioning that (and why) it is sufficient to express the exon 1 of mHTT1 only to induce aggregation.

Point-by-point response to the referees' comments

Referee #1:

EMBOJ-2020-107260
In this manuscript, Blumenstock et al extended previous studies from Firefly luciferase protein and its capacity as a sensor to monitor proteostasis capacity, now in this case in the central nervous system. Here, a new reporter mice carrying EGFP-fused firefly luciferase (Fluc-EGFP) is developed. The overexpressed product is an unstable protein that demands chaperone function for proper folding and reacts to proteotoxic stress by forming intracellular foci and reduced luciferase activity. Authors provide evidence of neuronal expression for this construct in response to the Prp promoter and evaluate the activation of the FLuc-EGFP system evaluating their results as proteostasis impairment in both tauopathy and Huntington's mice models. Overall, this manuscript is logic and straightforward, while it approaches properly to the mouse model and the function of the proposed system. However, a major concern along the data presented for this new transgenic mice model is the absence of key controls that demonstrate the establishment of the proteostasis impairment across figures. However, we believe that authors can easily solve this aspect. In addition, minor aspects in data or the interpretations are discussed. The idea of Fluc-GFP as a proteostasis monitor tool is a general concept and not a novelty in the field; however, this work proposes a novel animal model to monitor proteostasis capacity in the central nervous system. I list below major and minor points to improve the study, our requirements for publishing in EMBO Journal.

We thank the reviewer for appreciating our approach, and for the constructive suggestions.

1. Western blot against proteotoxic stress controls are required as supplementary information in order to demonstrate the appropriate induction in response to every treatment for Fig. 1A (positive controls after MG-132, bafilomycin, 17-AAG and heat shock). Protein synthesis, autophagy markers, Hsp90 activity and HSP activation should be shown in some way respectively.

As suggested by the reviewer, we performed immunoblots to demonstrate the efficiency of the applied inhibitors. For MG-132, we observed a clear increase in ubiquitinated proteins (Appendix Fig. S1A). For the Bafilomycin A1 treatment, we quantified the levels of LC3B-II and observed a significant increase (Appendix Fig. S1B).

To demonstrate a reduction in Hsp-90 activity in the presence of 17-AAG, we have tried detecting Hsf1 and p-Hfs1 with several available antibodies. As we did not manage to obtain a reliable signal with any of those antibodies, we resorted to an alternative readout, quantifying Akt phosphorylation, which has been shown to decrease after 24 hours of 17-AAG treatment (e.g., Chen et al., 2020, PMID 32319654). We detected a modest, but statistically significant decrease in p-Akt / Akt ratio, suggestive of Hsp90 inhibition (Appendix Fig. S1C).

To demonstrate heat shock induction, we used a panel of antibodies against heat shock proteins, including Hsp27, HspA6, Hsp70B, and Hsp70/72. Unfortunately, none of the antibodies we tried produced a reliable signal in neurons, despite having been used successfully in other cell types. Although we were not able to perform this control

experiment, we note that heat shock represents a very well-established paradigm to induce protein misfolding in various cells including primary neurons, and we have added several citations on this subject (Morimoto, 2011; Nishimura et al., 1991; Yang et al., 2008; see Results, p. 4).

As we have repeated some of the experiments shown in Fig. 1 and EV1 in order to present these control blots, we were also able to add new data points to one of the graphs. In particular, the increase in the fraction of cells with Fluc-EGFP foci upon treatment with MG-132 shown in Fig. EV1C is now statistically significant.

2. It has been reported that prion promoter activity decreases with aging in frontal cortex and hippocampal neurons (and indeed this doesn't happen in familial Alzheimer's disease, Whitehouse et al., 2010 J Alzheimers Dis). Protein abundance experiments are not as clear/clean as expected, regarding variability in the housekeeping control and low experimental number (western blots show only 3 samples per group). To solve this, please confirm the abundance of the housekeeping control with another control protein (GAPDH?) by western blot and also, aging experiment must be compared to Fluc-EGFP mRNA levels (a correlation study between Fluc-EGFP mRNA levels and luciferase activity also is an option). Furthermore, the comparison of wildtype littermates in the western blot is needed as basic evidence of this new transgenic mouse model.

All the aging groups consisted of 5 samples (as indicated on the bar graphs). We only show part of the samples in Fig. 2D in order not to overload the figure. The full unmodified blot is now provided as Source Data for Fig. 2D.

To overcome the issue of variability in the levels of individual housekeeping genes, we have used total protein for normalization of Western blots. These quantifications are shown in Appendix Fig. S2B. The results are overall very similar to the ones obtained with tubulin as a loading control (Fig. 2F).

As we did not have tissue samples suitable for RNA work available in the time frame of the revisions, we unfortunately could not quantify mRNA levels in Fluc-EGFP brains. Even though downregulation of prion protein (PrP) expression has been described in aging human brain as pointed out by the reviewer, this decrease might not happen in other species. In fact, another study reported an age-dependent increase in PrP protein levels in the mouse brain (Williams et al., 2004, PMID 15043713). Our protein quantification, despite variability in the data, does not reveal any marked changes in transgene expression in the Fluc-EGFP line over the life span of the mice (Fig. 2F and Appendix Fig. S2C).

As proposed by the reviewer, we have included samples from wildtype (Fluc-EGFP$^-$) littermates into the Western blot in Fig. 2C. This experiment demonstrates the specificity of the EGFP signal on the Western blot.

3. In addition, experiments of luciferase activity in Figure 2 lack of control from wildtype mice, since this is the first approach of this model.

As recommended by the reviewer, we have added luciferase activity measurements from brain lysates of non-transgenic littermates. We observed extremely low levels of

background luciferase activity in the absence of the transgene. The data is described in the Results on p. 6 and shown in Fig. EV2E.

4. I think that the sentence "...suggesting that only cytoplasmic protein aggregates cause a disturbance of neuronal proteostasis" is not close enough to be proposed as a suggestion with the available data showed here. The suggestion maybe also: this protein is unable to form nuclear inclusions due to a technical issue (for example, a difficulty to translocate). For example, under the cellular Htt-97Q model (nuclear IB), the FlucSM-EGFP sensor is able to detect nuclear inclusion bodies, whereas Fluc-GFP is not (Gupta et al., 2011 Nat Methods). In addition, this mutant Huntingtin model shows signatures of ER stress, both in cell lines and in mouse model (Hyrskyluoto et al., 2014 Hum Mol Gen) confirming proteostasis alterations. The sentence proposed is overstated and I suggest adjusting it to the presented evidence. In addition, I suggest that intracellular compartments studies must be performed. For example, the re-distribution of NLS-Fluc into the cytosol reveals that there may be some modifications of the protein that does not allow its translocation. In addition, the subtitle "Cytoplasmic, but not nuclear, protein aggregates cause proteostasis impairments" is overstated and could be replaced for "Cytoplasmic, but not nuclear, protein aggregates are detected by Fluc-EGFP reporter".

We do observe NLS-Fluc-EGFP foci in the nucleus (see quantification in Fig. 5E), suggesting that the sensor is able to translocate to the nucleus and to form foci in this compartment. However, we agree with the reviewer that the sensitivity of the reporter to proteostasis alterations in the nucleus vs. cytoplasm might be different. As proposed by both Reviewers #1 and #2, we have performed further compartment studies with another aggregating protein, β23. In this case we observed Fluc foci only in the cytoplasm, but not in the nucleus. These data, shown in Fig. EV5 and described on p. 10, are indeed compatible with the hypothesis that the sensor might be more suitable for detecting proteostasis disturbances in the cytoplasm than in the nucleus. We have therefore modified the respective statements throughout the paper as follows:

- Title: “Novel reporter mouse reveals neuronal proteostasis alterations in aging and disease”.
- Abstract: “Moreover, we find a marked reaction of the sensor in tauopathy mice, but not in Huntington’s disease mice. Mechanistic investigations in primary neuronal cultures demonstrate that different protein aggregates have distinct effects on the cellular protein quality control.”
- Subtitle in the Results (p.10): “Cellular compartment-specific reactions of Fluc-EGFP to different aggregating proteins”.
- Results, p. 10: “In summary, the Fluc-EGFP sensor reacts only to the presence of cytoplasmic, but not nuclear, mHTT aggregates, suggesting that cytoplasmic mHTT aggregates might cause a greater disturbance of neuronal proteostasis.” … “Taken together, these results suggest that different aggregating proteins cause distinct compartment-specific proteostasis impairments.” The first sentence is toned down and only refers to mHTT, not aggregates in general. Of note, a recent study based on a different proteostasis sensor with a FRET readout, also describes reaction of both nuclear and cytoplasmic sensor versions to cytoplasmic mHTT inclusion bodies (Raeburn et al., bioRxiv 2021, doi: https://doi.org/10.1101/2021.04.19.440383; citation included in the discussion on p. 13), providing an independent confirmation of some of our findings.

- Discussion, p. 12: "Our mechanistic investigations in cultured neurons suggest that both the nature of the aggregates as well as their subcellular localization might contribute to the differences in proteostasis between disease models. We observed that β23 aggregates had an effect on the solubility of cytoplasmic Fluc-EGFP, regardless of where the aggregates themselves were localized, while mHTT-exon1 IBs only induced Fluc reaction when they were localized in the cytoplasm. … While we cannot exclude differences in the sensitivity of the Fluc-EGFP sensor to proteostasis impairments in different cellular compartments, our results support the idea that nuclear and cytoplasmic compartments differ in their capacity to cope with protein aggregation."
- Discussion, last paragraph: "With the help of these mice, we uncovered unexpected differences in the impact of different aggregating proteins on the cellular protein quality control system."

5. The model proposes changes in the folding stability of Fluc-EGFP due to proteostasis impairment across the figures. However, as the results are presented, the absence of Fluc-EGFP inclusions does not mean so. Several other possibilities may occur such as the decrease of fluorescent signal or even conformational changes of Fluc protein and furthermore changes in luciferase activity may occur. A major concern throughout the data presented here is a general lack of proteostasis stress readouts elucidating such alterations. If this proposal is true, a proteostasis reestablishment experiment should solve this question. For example, a rescue experiment with chaperones overexpression (or by using chemical chaperones) should reestablish the correct folding/activity of Fluc-EGFP in proteostasis alterations context. Without this experiment, this article may be published in a methodologic journal. In my opinion, and for the interest of the Editor, at least one in vitro and one in vivo proposal of proteostasis rescue must be demonstrated here for publishing in EMBO Journal.

To test whether Fluc-EGFP response is dependent on proteostasis, we have performed proteostasis reestablishment experiments with a chemical chaperone, as proposed by the reviewer. We have taken two approaches, one *in vitro* in dissociated neurons, and one *ex vivo* in acute brain slices, using two different kinds of proteotoxic stress (mHTT as an aggregating protein, and heat shock, respectively), and two different readouts for Fluc-EGFP folding (Fluc-EGFP inclusion formation and luciferase activity, respectively). Brain slices were used as an alternative to an *in vivo* approach, because experiments in mice were not feasible within the time frame of the revisions.

In the *in vitro* experiments, we co-transfected dissociated neuronal cultures with Fluc-EGFP and mHTT-mCherry, and treated the neurons with the chemical chaperone 4-phenylbutyrate (4-PBA). While in the absence of 4-PBA, mHTT caused a significant increase in Fluc-EGFP foci, no significant change was observed in 4-PBA-treated cultures (Fig. 1D-E and text on p. 5).

In the *ex vivo* experiments, we pre-treated Fluc-EGFP brain slices with 4-PBA and subjected them to heat shock. Heat shock led to a clear reduction in Fluc-EGFP specific luciferase activity. This reduction was significantly ameliorated in the 4-PBA treated samples (Fig. 2C and text on p. 6). Taken together, these new findings indicate that Fluc-EGFP response in neurons is dependent on protein folding.

Referee #2:

General

The manuscript by Blumenstock et al describes the in vivo use of a well-known proteostasis reporter construct. A transgenic mouse line is constructed and crossed with 2 models of neurodegeneration, a tau line and a Huntington line, and the behaviour of the reporter in both is compared. Given the marked difference in response of the reporter in both models, the authors move to a cell-culture based comparison of cytoplasmic and nuclear aggregates, from which they conclude that nuclear aggregates are in general less burdensome for the proteostasis network.

The work is very interesting and the use of the reporter in vivo in models of aggregation disease is highly relevant, so I am supportive of publication. I have however a number of concerns that I think should be cleared up.

We thank the reviewer for the positive and supportive comments.

Main concerns

1. The proteostasis network is notoriously highly adaptive and thus the use of a transgenic line where the sensor is present during development is potentially fraught with complications since a new homeostatic point may be reached through an adaptive response that incorporates the sensor (itself a burden). I would be much less worried if an inducible construct had been used, switched on just before readout. Given that the model is what it is, can the authors provide data to show that this is not a concern, or otherwise include clear disclaimers to this potential pitfall? I am worried that if widely used in the field, it may generate red herrings.

We agree with the reviewer that expression of an unstable protein might result in adaptive changes in the proteostasis network. To address this concern, we have included new data assessing the magnitude of a stress response in Fluc-EGFP vs. control brain tissue. To this end, acute brain slices from Fluc-EGFP mice and wildtype littermates were subjected to proteotoxic stress by treating them with the proteasome inhibitor MG-132, and the resulting increase in ubiquitinated proteins was analyzed. We observed a similar increase in ubiquitination in slices of both genotypes (Fig. EV2F-G, and text on p. 6), arguing that the capacity for stress responses is not altered in the tissue from Fluc-EGFP mice. In addition, we have attempted analyzing the response of Fluc-EGFP brain slices to heat shock by blotting against a panel of heat shock proteins, but these analyses were precluded by the poor signal of all the used antibodies in brain tissue.

We have included a statement acknowledging the mentioned limitation, and explaining how this concern is mitigated in our mice (Discussion, p. 11): “It should be kept in mind that the expression of an unstable protein such as Fluc might itself impose a burden on the cellular quality control machinery and lead to long-term changes in the proteostasis network. In our transgenic line, the sensor is expressed at a mild level, mitigating potential adaptive changes. In addition, our experiments with brain slices (Fig. 2C), along with previous investigations in HeLa cells (Gupta et al., 2011), showed that Fluc-EGFP only has a minor effect on proteostasis, and does not alter cellular stress responses.”

2. The authors generalise the difference in response between HTT and Tau, to a general difference between aggregates in the nuclear and cytoplasmic compartments. However, the sequence composition flavour of both proteins is also completely different and I dont think that the cell culture work done is conclusive to support these claims. Cultured cells have everything in abundance and probably dont model proteostatic aging very well at all, we don't know what would happen with other proteins in nuclear aggregates in a mouse model over time and we cannot infer this from cell culture work. This part of the conclusion should at least be toned down in the abstract I think.

Can the authors exclude that the sensor is simply more sensitive to cytoplasmic perturbations? or that there is a delay before nuclear stress is picked up by the sensor (older age or in cell culture terms, longer incubation times)? How does the sensor respond to p53 aggregation eg. or something in the ER, like CFTR?

We agree with the reviewer that the nature of HTT and tau aggregates is quite different, and also that comparisons between cell culture and *in vivo* conditions are difficult. As recommended by the reviewer, we have performed compartment studies with another aggregating protein, β23. In these experiments, we observed Fluc foci in the cytoplasm, but not in the nucleus, regardless of where β23 was localized. These data, shown in Fig. EV5 and described on p. 10-11, are indeed compatible with the hypothesis that the sensor might be more suitable for detecting proteostasis disturbances in the cytoplasm than in the nucleus. We have therefore modified the respective statements throughout the manuscript:

- Title: "Novel reporter mouse reveals neuronal proteostasis alterations in aging and disease".
- Abstract: "Moreover, we find a marked reaction of the sensor in tauopathy mice, but not in Huntington's disease mice. Mechanistic investigations in primary neuronal cultures demonstrate that different protein aggregates have distinct effects on the cellular protein quality control."
- Subtitle in the Results (p.10): "Cellular compartment-specific reactions of Fluc-EGFP to different aggregating proteins".
- Results, p. 10: "In summary, the Fluc-EGFP sensor reacts only to the presence of cytoplasmic, but not nuclear, mHTT aggregates, suggesting that cytoplasmic mHTT aggregates might cause a greater disturbance of neuronal proteostasis." … "Taken together, these results suggest that different aggregating proteins cause distinct compartment-specific proteostasis impairments." The first sentence is toned down and only refers to mHTT, not aggregates in general. Of note, a recent study based on a different proteostasis sensor with a FRET readout, also describes reaction of both nuclear and cytoplasmic sensor versions to cytoplasmic mHTT inclusion bodies (Raeburn et al., bioRxiv 2021, doi: https://doi.org/10.1101/2021.04.19.440383; citation included in the discussion on p. 13), providing an independent confirmation of some of our findings.
- Discussion, p. 12: "Our mechanistic investigations in cultured neurons suggest that both the nature of the aggregates as well as their subcellular localization might contribute to the differences in proteostasis between disease models. We observed that β23 aggregates had an effect on the solubility of cytoplasmic Fluc-EGFP, regardless of where the aggregates themselves were localized, while mHTT-exon1 IBs only induced Fluc reaction when they were localized in the cytoplasm. … While

we cannot exclude differences in the sensitivity of the Fluc-EGFP sensor to proteostasis impairments in different cellular compartments, our results support the idea that nuclear and cytoplasmic compartments differ in their capacity to cope with protein aggregation."

- Discussion, last paragraph: "With the help of these mice, we uncovered unexpected differences in the impact of different aggregating proteins on the cellular protein quality control system."

Specific comments:

- In the tau model: the sensor is positive in the absence of tau hyperphosphorylation: the authors suggest this is because the proteostatic burden precedes the formation of insoluble aggregates and I think this is plausible, but it would be nice to have more certainty. For example: is there an increased staining for pFTAA in these cells? (or a similar sensor, they are avialable with a wide range of spectral properties)

We thank the reviewer for the helpful suggestion. To address this question, which was also mentioned by Reviewer #3, we have performed co-immunostainings with antibodies against total human tau (HT7) and phosphorylated tau (AT8). Interestingly, we observed that the fraction of cells with Fluc-EGFP foci, as well as the relative fluorescence intensity of the foci were increased to the same extent in HT7 cells that do and do not contain phosphorylated tau, while in HT7-negative cells it was not different from control mice without tau transgene expression (Fig. 3A-D, and text on p. 7-8). These data show that Fluc-EGFP forms foci in response to human mutant tau, even in the absence of mature tau tangles.

-Figure 2 panels C-D; E-F and G-H:
I think it would be a more correct way to analyse if you would normalise the luciferase activity to the protein concentration for each individual measurement. Actually, exactly how it was done in Figure 1C. On top of that, how is the protein quantity determined with a smear like in the conditions 12 mo 24 mo (2I), as it looks like there is quite a reduction in protein quantity in 12 mo, which is not visualised in the bar plot in Fig 2F. The same comment applies for Figures 3F-G and 4B-C.

Luciferase activity is already normalized to protein quantity throughout the paper, including the graphs in Fig. 3 and 4, exactly as in Fig. 1C. The normalized value is always referred to as "specific luciferase activity". To make this clear, we have modified the sentence on p. 4, where luciferase assay is described for the first time. It now reads "Throughout the study, luciferase activity measurements were normalized to Fluc-EGFP protein quantity determined by Western blot to obtain specific activity values."

As mentioned in the Materials and Methods, the entire background-adjusted lane area above the Fluc-EGFP monomer band was quantified to take potential high-molecular weight smear of Fluc signal into account. To make it clear, we have now indicated this area with a bracket next to the blots in Fig. 2D and EV3B.

Although the Fluc-EGFP monomer band appears reduced at 12 months in Fig. 2D, the tubulin band is also weaker than e.g. at 24 months. Together with the appearance of the

high molecular weight smear that was also quantified, this in the end results in comparable Fluc-EGFP protein quantities at 12 months as in other age groups.

- Figure 5
An obvious extra control would be to target the p-tau from rTg4510 mice to the nucleus. The sensor should not be detecting those inclusions. If it does, it is not the compartment that matters but the identity of the aggregate.

We agree the with reviewer's argument. To address this possibility, we have performed cellular compartment studies with an unrelated aggregating protein, β23-mCherry. We have also modified all the relevant statements in the text (see response to main concern 2 above).

- Final sentence of results on page 9: "In summary, the Fluc-EGFP sensor reacts only to the presence of cytoplasmic, but not nuclear, protein aggregates, suggesting that only cytoplasmic protein aggregates cause a disturbance of neuronal proteostasis."
The authors use one specific reporter here (Fluc), which, as stated, depends on chaperones for proper folding etc. The final statement made by the authors might be too general in the sense that if another sensor would be used, that depends on another set of chaperones, it might be possible to detect nuclear aggregate stress as well? Stating that only cytoplasmic protein aggregates cause a disturbance of neuronal proteostasis might be too broad?
In the same line: a sentence in the discussion: "proteostasis was largely intact in HD models". Not strictly correct: the reporter line does not detect it, which does not mean it's not there.

We agree with the reviewer and have modified these statements. The mentioned statement in the Results (p. 10) now refers only to mHTT and reads: "In summary, the Fluc-EGFP sensor reacts only to the presence of cytoplasmic, but not nuclear, mHTT aggregates, suggesting that cytoplasmic mHTT aggregates might cause a greater disturbance of neuronal proteostasis". As we have obtained a different result with another aggregating protein, we now conclude the Results with "Taken together, these results suggest that different aggregating proteins cause distinct compartment-specific proteostasis impairments". The mentioned sentence in the Discussion (p. 12) now reads: "In contrast to tauopathy mice, the Fluc-EGFP sensor did not show any reaction in HD models".

Minor concerns and suggestions

Given the widespread availability of single cells RNA sequencing techniques, I think the big opportunity in these models would be to analyse the cell-type specificity of the cells in which the reporter shows proteostatic decline.

This is indeed an interesting direction for further studies. We have added a statement about this to the Discussion (p. 12): "Combined with recent developments in single-cell RNA sequencing techniques, our reporter mouse offers an experimental tool to uncover the molecular basis of proteostasis differences between various cell types, by comparing the transcriptional signatures of cells with proteostasis differences revealed by the reporter".

What would really drive the adoption of this reporter in the field would be an AAV based

construct with a brain tropism or other that can be introduced in any neurodegenerative disease model at any desired age. This would also overcome the issue of adaptive responses.

We agree with the reviewer and have added the following sentence to the Discussion (p. 11): "…Using the sensor in the context of inducible genetic models and viral-based strategies is an exciting possibility for future studies that would further minimize this limitation."

Referee #3:

EMBOJ-2020-107260 review.20201210
Proteostasis is known to be impaired in neurodegenerative diseases generating a vicious cycle by which the protein quality control system is being overloaded by protein aggregates. Here, a luciferase read-out of cytoplasmic aggregation and reduced activity (wild-type and aggregation-prone mutant) was tested in vitro followed by generating transgenic mice driven by the PrP promoter. Decline in luciferase activity was found with ageing. By crossing the parental strain onto tau and HTT mutant backgrounds it was found that tau (being cytoplasmic) impaired the proteosome (reporter) whereas huntingtin (being nuclear) did not. Readouts were luciferase foci and reduced bioluminescence. A cross-over design in cell culture then showed that cytoplasmically localized Htt is capable of impairing the proteosome. The authors conclude (as stated in the abstract) that cytoplasmic, but not nuclear aggregates cause defects of cellular protein quality control.

The introduction is concise focusing on what is relevant for this particular study. The results section however lacks critical information such as age groups studied, brain areas and cell-types analyzed etc. I consider the data as interesting and the tools advancing the field, but the in vivo analysis needs to be worked over as outlined below. The discussion should go into the limitations around the in vivo models (see below).

We thank the reviewer for pointing out the strengths of the paper, and for providing constructive suggestions.

Page 5/Fig 2A,B: Histological analysis of parental transgenic strain (PrP-FlucWT-EGFP): What is meant by salt-and-pepper-like distribution of expression? The transgenic product seems to be distributed into the soma and dendrites, exclude the nucleus, and expression is particularly strong in cortex and hippocampus. Is there any glial expression (as one would expect for the PrP promoter)?

We have modified the description of the transgene expression following reviewer's suggestions. To characterize expression in neurons vs. glia, we have furthermore performed co-immunostainings with a neuronal marker (Neurotrace) and glial markers: GFAP for astrocytes, APC for oligodendrocytes, and Iba1 for microglia. These analyses revealed that Fluc-EGFP was restricted to neurons and not detectable in any of the glial cell types examined (Fig. EV2A and Appendix Fig. 2A). The respective text now reads (p. 5-6): "For further experiments, we selected the FlucWT-EGFP line 1214 (from here on, Fluc-EGFP mice), which showed a broad expression of the transgene throughout the brain, including regions affected in neurodegenerative proteinopathies. In particular, stronger expression was detected in the neocortex and hippocampus, while lower levels

were observed in the basal ganglia and cerebellum (Fig. 2A-B). Co-staining with cell type markers demonstrated that Fluc-EGFP was present in Neurotrace+ neurons, while it was not detectable in GFAP+ astrocytes, APC+ oligodendrocytes, or Iba+ microglia (Fig. EV2A and Appendix Fig. S2A). In neurons, Fluc-EGFP showed cytoplasmic localization in the soma and dendrites (Fig. 2B, EV2A and Appendix Fig. S2A)".

Page 6/Fig 2C-H: Decline of luciferase expression with ageing: I suggest swapping panels H and I as H is the quantification of I.

We have quantified Fluc-EGFP protein levels in three different brain regions, but show only one representative blot to avoid overloading the figure. The blot shows hippocampal lysates (i.e. F is the quantification of I according to the previous numbering of the panels in the figure). We have now redesigned this figure, and show the blot of hippocampal lysates before the respective quantification (Fig. 2D, F). We hope that the new arrangement and labeling of the panels is clear.

The blot (2I) needs to be repeated as this is a composite blot!

All the lanes shown in this panel do belong to the same blot, although we digitally removed a part of the blot between the 6 months and 12 months samples. The full unmodified blot is now provided as Source Data for Fig. 2D.

The loading control needs to be revisited: the tubulin signal at 24 mo is much stronger than at 12 mo which makes me sceptical about the increase in Fluc protein with ageing. The MW needs to be indicated and the blot should also be probed for Fluc. For the 12 mo samples some protein is stuck in the well. The pockets should be shown for all lanes. Also, it should be indicated on the blot what has been used for quantification. (same for Fig S3 where there is a HMW smear also for the control)

To overcome the issue of variability in the levels of individual housekeeping genes (also pointed out by reviewer #1, comment 2), we have used total protein (visualized by stain-free technology from Bio-Rad) for normalization of Western blots from the ageing experiments. This data is shown in Appendix Fig. S2B. Of note, the quantifications are very similar to the ones with tubulin as a loading control (Fig. 2F).

We have indicated the molecular weight at the left side of all the blots.

We have compared immunodetection of GFP and Luciferase on the same blot and found that, while the overall pattern of Fluc-EGFP bands was similar, the anti-GFP antibody produced a much more specific signal (Appendix Fig. S2A). For this reason, we used GFP detection for Western blots throughout the paper.

The image of the blot already includes the pockets for all the lanes.

Following the reviewer's suggestion, we have indicated the area that was used for quantification with a bracket next to the blots in Fig. 2D and EV3B.

Page 6/Fig 3: Crossing with rTg4510 tau mice: It is difficult to compare these data with the situation in the parental mouse strain in Fig 2 as a different magnification is shown, the nuclei

are not shown, and it is not even clear in which brain area the images have been captured. The AT8 distribution looks unfamiliar to me. I am surprised that the CA layer of the hippocampus has not been used, with its typical cytoarchitecture. I am not disputing that more foci form in the crossed triple tg mice but the data presentation is unfortunate.

We have included zoomed images of cells in Fig. 2B to make it more comparable to Fig. 3A. We have also replaced Neurotrace labeling in Fig. 3A with DAPI to show the nuclei. As indicated in the figure legend, the images were captured from cortical sections. We have indicated the brain region and age on the figure to make it clear. The new images in Fig. 3A show a more typical distribution of AT8 immunoreactivity.

The CA layer of the hippocampus is shown in the upper and lower row of images in Fig. EV3A. We have now marked it with a dashed line to make it clear.

In 3E the authors claim that the % of cells with EGFP foci of AT8+ phospho-tau cells is the same as in AT8- phospho-tau cells. They conclude that 'Fluc-EGFP is sensitive to protein aggregation in the cytoplasm prior to formation of insoluble aggregates'; however, the authors don't state (as far as I can tell) how old the mice were when the images were taken and whether the AT8- phospho-tau cells express tau at all. They could use a pan-human tau antibody to show.

The stainings were performed in 4-month-old mice. We have indicated the age on the figure.

We thank the reviewer for the helpful suggestion to use a pan-human tau antibody (a similar point was made by Reviewer #2, specific comment 1). We have performed co-immunostainings for pan-human tau (HT7) and phosphorylated tau (AT8). Interestingly, we find that the relative intensity of Fluc-EGFP foci, as well as the fraction of foci containing cells are increased to the same extent in HT7 cells that do and do not contain phosphorylated tau, while in HT7-negative cells they are not different from control mice without tau transgene expression (Fig. 3A-D, and text on p. 7-8). These data show that Fluc-EGFP reacts to human mutant tau, even in the absence of mature tau tangles.

Page 7/Fig 4: Please specify in legend 4 that EM48 detects huntingtin. Huntingtin accumulates in the nucleus. Does Huntingtin 'see' the proteosome? I understand that there is a lot of literature claiming and showing proteasomal impairment in HD and HD models. How is this being reconciled? The mice (2 models) were only 3 months old (or younger). Is it possible that the pathology is simply not advanced enough, especially as for tau pathology a model was chosen that has been challenged because of a massive overexpression and an (FGF14) integration artefact. To me it is difficult to compare the tau and Htt models and the discussion should critically discuss the models and to which extent the data can be generalized. I understand that a lot work has gone into establishing these mice and am not expecting a second tau model being tested but the discussion should discuss the limitations.

It is already mentioned in legend 4 that EM48 detects aggregated mutant Huntingtin (p. 25): “Cortical (upper row) and striatal (lower row) sections from 12-week-old R6/2:Fluc-EGFP mice (right) and control WT:Fluc-EGFP littermates (left) stained for aggregated mHTT (EM48, magenta).”

We have expanded the paragraph in the Discussion that deals with proteasome defects in HD mice, mentioning the existing evidence of UPS impairment in HD and providing possible explanations to reconcile the different findings. This paragraph now reads (p. 12): "In contrast to tauopathy mice, the Fluc-EGFP sensor did not show any reaction in HD mice. … Our findings are in line with previous studies using UPS reporters, which demonstrated UPS impairment in the rTg4510, but not R6/2 model (Bett et al., 2009; Maynard et al., 2009; Myeku et al., 2016). As there is extensive evidence of UPS defects in HD, the negative results obtained with UPS sensors as well as with our sensor could be partially due to the long-term compensatory changes in mice with constitutive expression of mHTT (Ortega et al., 2010; Ortega and Lucas, 2014). However, the observations we made in the HD94 line are seemingly in contrast to a previous report, where accumulation of the UPS reporter Ub-G76V-GFP was detected upon acute induction of mHTT expression (Ortega et al., 2010). Of note, our results do not exclude the possibility that protein degradation by UPS might be impaired in HD94 mice, however, they suggest that other components of the protein quality control machinery may compensate for the UPS defect. "

The R6/2 line is a rapid model with a life span of only ~4 months. At the age of 3 months the mice already reach advanced disease stage with very abundant mHTT inclusions and severe behavioral symptoms. It therefore seems unlikely that the pathology in this line would be not advanced enough for detecting possible proteostasis alterations.

We agree with the reviewer that the HD and tauopathy models are difficult to compare, and acknowledged this in the Discussion by including the statement (p. 12): "As these disease models are based on different genetic strategies and have different overexpression levels of the respective pathogenic protein, comparisons between them should be made with caution."

Page 8/Fig 5 is the logical experiment that comes to mind after having seen the in vivo data. By targeting HTT to the cytoplasm proteostasis impairments are being induced. All controls were done of nuclear and cytoplasmic targeting of HTT and the reporter. As a minor point for someone not working in the Huntington's space it might be worthwhile mentioning that (and why) it is sufficient to express the exon 1 of mHTT1 only to induce aggregation.

We have included the requested sentence on p. 5: "mHTT-exon1 is a key pathogenic version of the protein that is sufficient to recapitulate HD phenotypes (Mangiarini et al., 1996; Sathasivam et al., 2013; Yang et al., 2020)."

We have also specified in the text that both HD models used in the study express mHTT-exon1. The text was modified as follows:

- "In addition to tauopathy mice, we investigated proteostasis in the R6/2 mouse model of HD. R6/2 is an early-onset transgenic model that expresses mHTT-exon1 under the human HTT promoter" (p. 8).
- "To test this, we crossed Fluc-EGFP mice to the inducible HD94 mouse line (CaMKIIα-tTA:BiTetO-HTT-Q94) (Yamamoto et al., 2000), which allows for precise temporal control over mHTT-exon1 expression" (p. 9).

1st Revision - Editorial Decision 28th Jun 2021

Dear Irina,

Thank you for submitting your revised manuscript to The EMBO Journal. Your study has now been seen by the three referees. As you can see below, the referees appreciate the introduced changes and support publication here. I am therefore very pleased to let you know that we will accept your manuscript for publication here.

Before I can send you the accept letter, there are just a few editorial things to sort out. You can use the link below to upload the revised version

- You can only have 5 keywords

- For the checklist please add not applicable to all the sections that are not relevant like 13-17 and 19-21

- For the reference list: for articles with more than 10 authors the author list should be cut after 10 authors followed by et al.

We encourage the publication of source data, particularly for electrophoretic gels and blots, with the aim of making primary data more accessible and transparent to the reader. It would be great if you could provide me with a PDF file per figure that contains the original, uncropped and unprocessed scans of all or key gels used in the figure? The PDF files should be labelled with the appropriate figure/panel number and should have molecular weight markers; further annotation could be useful but is not essential. The PDF files will be published online with the article as supplementary "Source Data" files.

The size of synopsis is not correct should be 550 wide by [200-400] high (pixels)

I have asked our publisher to do their checks on the paper. They will send me the file within the next few days. Please wait to upload the revised version until you have received their comments.

That should be all. Let me know if you have any further questions.

With best wishes

Karin

Karin Dumstrei, PhD
Senior Editor
The EMBO Journal

Guide For Authors: https://www.embopress.org/page/journal/14602075/authorguide

The revision must be submitted online within 90 days; please click on the link below to submit the revision online before 26th Sep 2021.

Link Not Available

Referee #1:

I appreciate the response, in addition to other modifications included suggested by other reviewers. The final results are strong and generated a balanced article. I recommend this article for publishing in The EMBO Journal.

I do not have any other corrections to add.

Referee #2:

The authors have carefully addressed the points I raised in the initial review, I am fully supportive of publication.

Referee #3:

The authors have addressed all my concerns and provided a detailed and satisfying response to the points I had raised. (It might have helped though to mark the changes that have been made to the text.)

The authors performed the requested editorial changes.

2nd Revision - Editorial Decision 19th Jul 2021

Dear Irina,

Thank you for submitting your revised manuscript to The EMBO Journal. I have now had a chance to take a close look at everything and all looks good.

I am therefore very pleased to accept the manuscript for publication here.

With best wishes

Karin

Karin Dumstrei, PhD
Senior Editor
The EMBO Journal

Please note that it is EMBO Journal policy for the transcript of the editorial process (containing referee reports and your response letter) to be published as an online supplement to each paper. If you do NOT want this, you will need to inform the Editorial Office via email immediately. More information is available here:
https://www.embopress.org/page/journal/14602075/authorguide#transparentprocess

Your manuscript will be processed for publication in the journal by EMBO Press. Manuscripts in the PDF and electronic editions of The EMBO Journal will be copy edited, and you will be provided with page proofs prior to publication. Please note that supplementary information is not included in the proofs.

Please note that you will be contacted by Wiley Author Services to complete licensing and payment information. The 'Page Charges Authorization Form' is available here:
https://www.embopress.org/pb-assets/embo-site/tej_apc.pdf

Should you be planning a Press Release on your article, please get in contact with embojournal@wiley.com as early as possible, in order to coordinate publication and release dates.

If you have any questions, please do not hesitate to call or email the Editorial Office. Thank you for your contribution to The EMBO Journal.

EMBO PRESS

YOU MUST COMPLETE ALL CELLS WITH A PINK BACKGROUND ➔

PLEASE NOTE THAT THIS CHECKLIST WILL BE PUBLISHED ALONGSIDE YOUR PAPER

Corresponding Author Name: Irina Dudanova
Journal Submitted to: The EMBO Journal
Manuscript Number: EMBOJ-2020-107260R

Reporting Checklist For Life Sciences Articles (Rev. June 2017)

This checklist is used to ensure good reporting standards and to improve the reproducibility of published results. These guidelines are consistent with the Principles and Guidelines for Reporting Preclinical Research issued by the NIH in 2014. Please follow the journal's authorship guidelines in preparing your manuscript.

A- Figures

1. Data

The data shown in figures should satisfy the following conditions:

- ➔ the data were obtained and processed according to the field's best practice and are presented to reflect the results of the experiments in an accurate and unbiased manner.
- ➔ figure panels include only data points, measurements or observations that can be compared to each other in a scientifically meaningful way.
- ➔ graphs include clearly labeled error bars for independent experiments and sample sizes. Unless justified, error bars should not be shown for technical replicates.
- ➔ if n< 5, the individual data points from each experiment should be plotted and any statistical test employed should be justified
- ➔ Source Data should be included to report the data underlying graphs. Please follow the guidelines set out in the author ship guidelines on Data Presentation.

2. Captions

Each figure caption should contain the following information, for each panel where they are relevant:

- ➔ a specification of the experimental system investigated (eg cell line, species name).
- ➔ the assay(s) and method(s) used to carry out the reported observations and measurements
- ➔ an explicit mention of the biological and chemical entity(ies) that are being measured.
- ➔ an explicit mention of the biological and chemical entity(ies) that are altered/varied/perturbed in a controlled manner.

- ➔ the exact sample size (n) for each experimental group/condition, given as a number, not a range;
- ➔ a description of the sample collection allowing the reader to understand whether the samples represent technical or biological replicates (including how many animals, litters, cultures, etc.).
- ➔ a statement of how many times the experiment shown was independently replicated in the laboratory.
- ➔ definitions of statistical methods and measures:
  - common tests, such as t-test (please specify whether paired vs. unpaired), simple $\chi 2$ tests, Wilcoxon and Mann-Whitney tests, can be unambiguously identified by name only, but more complex techniques should be described in the methods section;
  - are tests one-sided or two-sided?
  - are there adjustments for multiple comparisons?
  - exact statistical test results, e.g., P values = x but not P values < x;
  - definition of 'center values' as median or average;
  - definition of error bars as s.d. or s.e.m.

Any descriptions too long for the figure legend should be included in the methods section and/or with the source data.

In the pink boxes below, please ensure that the answers to the following questions are reported in the manuscript itself. Every question should be answered. If the question is not relevant to your research, please write NA (non applicable). We encourage you to include a specific subsection in the methods section for statistics, reagents, animal models and human subjects.

USEFUL LINKS FOR COMPLETING THIS FORM

http://www.antibodypedia.com
http://1degreebio.org
http://www.equator-network.org/reporting-guidelines/improving-bioscience-research-repor
http://grants.nih.gov/grants/olaw/olaw.htm
http://www.mrc.ac.uk/Ourresearch/Ethicsresearchguidance/Useofanimals/index.htm
http://ClinicalTrials.gov
http://www.consort-statement.org
http://www.consort-statement.org/checklists/view/32-consort/66-title
http://www.equator-network.org/reporting-guidelines/reporting-recommendations-for-tumo
http://datadryad.org
http://figshare.com
http://www.ncbi.nlm.nih.gov/gap
http://www.ebi.ac.uk/ega
http://biomodels.net/
http://biomodels.net/miriam/
http://jjj.biochem.sun.ac.za
http://oba.od.nih.gov/biosecurity/biosecurity_documents.html
http://www.selectagents.gov/

B- Statistics and general methods

Please fill out these boxes ➔ (Do not worry if you cannot see all your text once you press return)

1.a. How was the sample size chosen to ensure adequate power to detect a pre-specified effect size?	Materials and Methods: section "Statistical Analysis". The effect size was not pre-specified. Sample size is indicated for all experiments in the corresponding figure legends and was chosen according to our previous experience with neurodegenerative disease mouse models.
1.b. For animal studies, include a statement about sample size estimate even if no statistical methods were used.	Materials and Methods: section "Statistical Analysis". The sample size of animals was chosen according to our previous experience with neurodegenerative disease mouse models.
2. Describe inclusion/exclusion criteria if samples or animals were excluded from the analysis. Were the criteria pre-established?	All results include the entire datasets without exclusions.
3. Were any steps taken to minimize the effects of subjective bias when allocating animals/samples to treatment (e.g. randomization procedure)? If yes, please describe.	No specific randomization was used.
For animal studies, include a statement about randomization even if no randomization was used.	Materials and Methods: section "Mice". No specific randomization was performed.
4.a. Were any steps taken to minimize the effects of subjective bias during group allocation or/and when assessing results (e.g. blinding of the investigator)? If yes please describe.	Materials and Methods: section "Immunofluorescence". Wherever possible, the investigator was blinded to the treatment of primary neurons in order to assess the reaction of the Fluc-EGFP sensor.
4.b. For animal studies, include a statement about blinding even if no blinding was done	Materials and Methods: section "Mice". No specific blinding was performed.
5. For every figure, are statistical tests justified as appropriate?	Yes. Materials and Methods: section "Statistical Analysis".
Do the data meet the assumptions of the tests (e.g., normal distribution)? Describe any methods used to assess it.	Yes. Materials and Methods: section "Statistical Analysis".
Is there an estimate of variation within each group of data?	Yes. Materials and Methods: section "Statistical Analysis".

Is the variance similar between the groups that are being statistically compared?	Yes. Materials and Methods: section "Statistical Analysis".

C- Reagents

6. To show that antibodies were profiled for use in the system under study (assay and species), provide a citation, catalog number and/or clone number, supplementary information or reference to an antibody validation profile. e.g., Antibodypedia (see link list at top right), 1DegreeBio (see link list at top right).	Materials and Methods: section "Immunofluorescence" and "Luciferase assay and Western blotting"
7. Identify the source of cell lines and report if they were recently authenticated (e.g., by STR profiling) and tested for mycoplasma contamination.	Not applicable

* for all hyperlinks, please see the table at the top right of the document

D- Animal Models

8. Report species, strain, gender, age of animals and genetic modification status where applicable. Please detail housing and husbandry conditions and the source of animals.	Materials and Methods: section "Mice". Mice (Mus musculus) of both genders were used, age and strain as specified throughout the manuscript.
9. For experiments involving live vertebrates, include a statement of compliance with ethical regulations and identify the committee(s) approving the experiments.	Materials and Methods: section "Mice". All animal experiments were approved by the Government of Upper Bavaria (animal protocols 55.2-1-54-2532-13-13 and 55.2-1-54-2532-168-14)
10. We recommend consulting the ARRIVE guidelines (see link list at top right) (PLoS Biol. 8(6), e1000412, 2010) to ensure that other relevant aspects of animal studies are adequately reported. See author guidelines, under 'Reporting Guidelines'. See also: NIH (see link list at top right) and MRC (see link list at top right) recommendations. Please confirm compliance.	Materials and Methods: section "Mice". We confirm to comply with the ARRIVE guidelines

E- Human Subjects

11. Identify the committee(s) approving the study protocol.	Not applicable
12. Include a statement confirming that informed consent was obtained from all subjects and that the experiments conformed to the principles set out in the WMA Declaration of Helsinki and the Department of Health and Human Services Belmont Report.	Not applicable
13. For publication of patient photos, include a statement confirming that consent to publish was obtained.	Not applicable
14. Report any restrictions on the availability (and/or on the use) of human data or samples.	Not applicable
15. Report the clinical trial registration number (at ClinicalTrials.gov or equivalent), where applicable.	Not applicable
16. For phase II and III randomized controlled trials, please refer to the CONSORT flow diagram (see link list at top right) and submit the CONSORT checklist (see link list at top right) with your submission. See author guidelines, under 'Reporting Guidelines'. Please confirm you have submitted this list.	Not applicable
17. For tumor marker prognostic studies, we recommend that you follow the REMARK reporting guidelines (see link list at top right). See author guidelines, under 'Reporting Guidelines'. Please confirm you have followed these guidelines.	Not applicable

F- Data Accessibility

18: Provide a "Data Availability" section at the end of the Materials & Methods, listing the accession codes for data generated in this study and deposited in a public database (e.g. RNA-Seq data: Gene Expression Omnibus GSE39462, Proteomics data: PRIDE PXD000208 etc.) Please refer to our author guidelines for 'Data Deposition'.<br><br>Data deposition in a public repository is mandatory for:<br>a. Protein, DNA and RNA sequences<br>b. Macromolecular structures<br>c. Crystallographic data for small molecules<br>d. Functional genomics data<br>e. Proteomics and molecular interactions	"Data Availability" section is provided at the end of the Materials and Methods. This study includes no data deposited in external repositories.
19. Deposition is strongly recommended for any datasets that are central and integral to the study; please consider the journal's data policy. If no structured public repository exists for a given data type, we encourage the provision of datasets in the manuscript as a Supplementary Document (see author guidelines under 'Expanded View' or in unstructured repositories such as Dryad (see link list at top right) or Figshare (see link list at top right).	Not applicable
20. Access to human clinical and genomic datasets should be provided with as few restrictions as possible while respecting ethical obligations to the patients and relevant medical and legal issues. If practically possible and compatible with the individual consent agreement used in the study, such data should be deposited in one of the major public access-controlled repositories such as dbGAP (see link list at top right) or EGA (see link list at top right).	Not applicable
21. Computational models that are central and integral to a study should be shared without restrictions and provided in a machine-readable form. The relevant accession numbers or links should be provided. When possible, standardized format (SBML, CellML) should be used instead of scripts (e.g. MATLAB). Authors are strongly encouraged to follow the MIRIAM guidelines (see link list at top right) and deposit their model in a public database such as Biomodels (see link list at top right) or JWS Online (see link list at top right). If computer source code is provided with the paper, it should be deposited in a public repository or included in supplementary information.	Not applicable

G- Dual use research of concern

22. Could your study fall under dual use research restrictions? Please check biosecurity documents (see link list at top right) and list of select agents and toxins (APHIS/CDC) (see link list at top right). According to our biosecurity guidelines, provide a statement only if it could.	Not applicable
